# Biosimilars in the Era of Artificial Intelligence—International Regulations and the Use in Oncological Treatments

**DOI:** 10.3390/ph17070925

**Published:** 2024-07-10

**Authors:** Tomas Gabriel Bas, Vannessa Duarte

**Affiliations:** Escuela de Ciencias Empresariales, Universidad Católica del Norte, Coquimbo 1781421, Chile; vannessa.duarte@ucn.cl

**Keywords:** biosimilars, continents, regulations, oncology, cancer, FDA, EMA, artificial intelligence

## Abstract

This research is based on three fundamental aspects of successful biosimilar development in the challenging biopharmaceutical market. First, biosimilar regulations in eight selected countries: Japan, South Korea, the United States, Canada, Brazil, Argentina, Australia, and South Africa, represent the four continents. The regulatory aspects of the countries studied are analyzed, highlighting the challenges facing biosimilars, including their complex approval processes and the need for standardized regulatory guidelines. There is an inconsistency depending on whether the biosimilar is used in a developed or developing country. In the countries observed, biosimilars are considered excellent alternatives to patent-protected biological products for the treatment of chronic diseases. In the second aspect addressed, various analytical AI modeling methods (such as machine learning tools, reinforcement learning, supervised, unsupervised, and deep learning tools) were analyzed to observe patterns that lead to the prevalence of biosimilars used in cancer to model the behaviors of the most prominent active compounds with spectroscopy. Finally, an analysis of the use of active compounds of biosimilars used in cancer and approved by the FDA and EMA was proposed.

## 1. Introduction

The growing approval of biosimilar drugs, designed to precisely mimic their peers of biological origin, has generated some changes in the pharmaceutical industry. This fact occurs after the expiration of patents (intellectual property) that protect the inventions of molecules of biological origin from being copied for 20 years from their creation [1,2,3,4]. Protection of intellectual property causes a monopoly in favor of inventors and patent owners and therefore an increase in the cost of the law-protected medicine, making it less accessible to patients with fewer resources [5,6,7,8,9,10]. However, the expiration of patents opens up new opportunities for these biological molecules, enabling the creation of new medicines known as biosimilars, which must comply with rigorous regulatory conditions [11]. Many of these molecules have preliminarily had a long history of efficacy in the treatment of complex conditions, particularly autoimmune and oncological diseases [12,13,14]. In other words, with the expiration of these patents, the manufacture of biosimilars has become an increasingly profitable and accessible alternative to expensive original biological therapies [10,11,12,15,16,17,18]. The development and integration of biosimilars into healthcare systems present a unique set of challenges and opportunities, but unlike chemical-based generic drugs, biosimilars are not exact replicas of their original counterparts [19,20]. Biosimilars share a high degree of similarity in terms of protein sequence, efficacy, safety, and quality to the original biological molecule, but variations in the manufacturing process often lead to differences in the structure of the active protein [21,22]. Due to this complexity, a comprehensive understanding of biosimilars and very strict regulatory frameworks are required to ensure their safe and effective implementation equivalent to the original biological molecules [11].

This article explores the diverse regulatory landscape for biosimilars across eight countries spanning four continents: the United States, Canada, Australia, Japan, South Korea, Argentina, Brazil, and South Africa. These countries were selected for their varied regulatory approaches and significant influence on the global biosimilar market. The analysis delves into the regulatory policies and approval processes of each country, emphasizing the complexity of these processes and the necessity for standardized regulatory guidelines. It addresses inconsistencies in regulations, particularly regarding biosimilars developed, manufactured, and utilized in developed versus developing nations. Despite these variations, commonalities and differences exist in how biosimilars are regulated across the studied countries. For instance, in the United States, the FDA oversees biosimilar quality, whereas Health Canada fulfills this role in Canada. Both nations maintain stringent regulatory frameworks with detailed requirements to demonstrate biosimilarity, safety, and efficacy compared to original biological medicines [23,24,25,26]. In Australia, for its part, the Therapeutic Goods Administration (TGA) is the one that regulates everything related to biosimilars [27]. In Japan, supervision is carried out by the Pharmaceuticals and Medical Devices Agency (PMDA), which presents innovative and collaborative approaches for the evaluation of biosimilars [28]. In South Korea, the Ministry of Food and Drug Safety (MFDS) has emerged as a leader in the supervision and production of biosimilars, driving favorable policies to encourage research and development in this field [29,30,31]. In South America, Argentina and Brazil have adopted regulatory policies in line with the recommendations of the World Health Organization (WHO), but have also implemented specific regulations that consider their local contexts [12]. The National Administration of Medicines, Food, and Medical Technology (ANMAT) in Argentina and the National Health Surveillance Agency (ANVISA) in Brazil are working to balance the accessibility of these drugs with the safety and effectiveness of biosimilars [12,25,32,33]. South Africa, representing the African continent, faces unique challenges in terms of infrastructure and resources, but the South African Health Products Regulatory Authority (SAHPRA) is making progress in establishing regulations that allow the safe and effective introduction of biosimilars into its market, thus improving access to high-quality biological treatments at more reasonable costs [34]. Harmonizing these regulations, along with the incorporation of advanced technologies, such as artificial intelligence into research and development processes, is essential to ensure that biosimilars meet the quality, safety, and efficacy standards necessary to benefit patients around the world.

This leads to the second factor addressed in this research through an analysis of the use of artificial intelligence (AI) in the development of biosimilars, which would significantly improve the efficiency in time and quality of research and production of these medications [35,36]. AI is particularly used to optimize manufacturing processes, predict the stability of molecules, and improve bioequivalence studies in more urgent times [37]. Furthermore, it can help to quickly identify possible structural and functional variations of molecules, ensuring that biosimilars maintain the required quality and safety throughout the study [38]. Some AI tools typically used are ‘computational modeling and simulation’, which allow for predicting the behavior and stability of biological molecules under specific conditions [39]; ‘machine learning and deep learning’, which are techniques used to analyze large volumes of data from preclinical and clinical studies [40]; ‘predictive analytics’, which uses historical and current data, where predictive analysis algorithms can anticipate problems in production and supply chain, optimize logistics, and reduce costs [36]; ‘natural language processing (NLP)’, used to review and analyze large amounts of validated scientific literature, as well as patents, allowing researchers to stay up-to-date with the most recent advances and even identify possible intellectual property problems [41]; ‘convolutional neural networks (CNNs)’, through the analysis of biological images, CNNs can identify and classify cellular and molecular structures with great precision, facilitating quality control and comparison between biosimilars and their reference biologics [42].

Finally, this research proposes a third analysis, which is based on active compounds used in biosimilars and approved by the FDA and EMA for cancer treatment [43]. The interest in these molecules in particular results from the existence of a greater slowness than in other therapeutic areas for the integration of these biosimilars into oncological clinical practice, fundamentally due to the need for more solid clinical evidence, and in many cases, due to the preference that would exist due to the original medication from both patients and treating physicians [44,45]. The use of biosimilars in cancer treatment in the United States and Europe was prioritized due to the safety guaranteed by their regulations within the framework of the development of biosimilars in oncology, including exhaustive comparative studies and demonstrations of non-inferiority in terms of safety and effectiveness [46].

The research methodology was based on a systematic review of the most representative literature, which allowed us to compile a complete collection of documents related to the three aspects investigated [47]. That is, regulations in the eight countries of interest, the use of AI in the development of biosimilars, and biosimilars applied to cancer treatments in Europe and the United States [48].

## 2. Methodology

**Figure 1:**
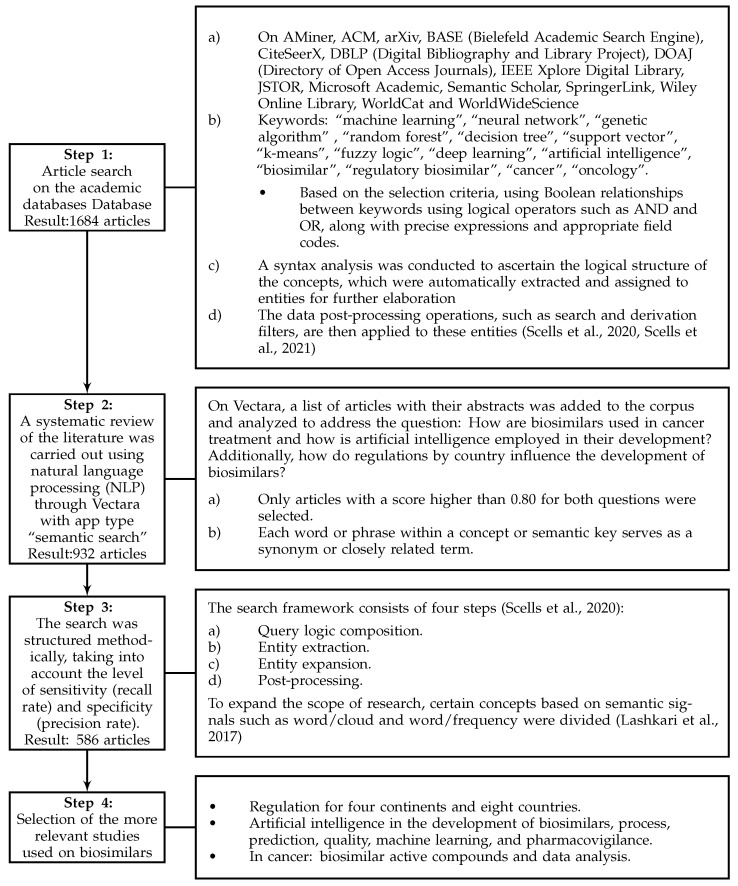
Methodology used in the study [50,51,52].

Figure 1 illustrates the steps taken to improve search effectiveness. Various documentary sources, bibliographic databases, and search engines tailored to each selected field or subject area were employed. These included Science Direct, Compendex, Derwent, Statistics Canada, Scopus, Web of Science Core Collections, Google Scholar, Innovation Index, and GeoIndex. Furthermore, interdisciplinary research tools were integrated to enhance the breadth of search systems [49].

During the initial phase, a comprehensive repository of 586 documents was collected, including research articles from journals indexed in Clarivate and Scopus, reviews, books, book chapters, and conference proceedings. It is important to note that this sample is representative of the information analyzed, but in no way is it intended to cover all existing documents related to the topic of the study ‘Biosimilars’ in the databases consulted.

## 3. Biosimilars: Characteristics and Perspectives

Emerging as a cornerstone in the field of biopharmaceuticals, biosimilars are meticulously designed complex bio-therapeutic products that mirror existing biological medicines, known as originator or reference products [19]. Unlike conventional small-molecule generics, these are large complex molecules that are typically derived from living cells, representing significant advancement and complexity in drug development [53]. At the molecular level, biosimilars share identical primary protein sequences with their reference drugs, and this similarity extends to their three-dimensional structures, crucial for biological activity [37]. However, the production of biosimilars introduces small differences in higher-order structures that are possible and significant, requiring extensive regulatory evaluation to ensure that the efficacy and safety profiles of biosimilars closely match those of the original biological products [5]. The physicochemical properties of biosimilars are a central point in their characterization, ensuring their alignment with those of the reference drug [54]. This involves a comprehensive evaluation of molecular weight, isoform patterns, impurity profiles, and other biochemical properties using advanced analytical methods [23]. These evaluations are crucial to confirm that any differences do not affect the clinical performance of the biosimilar. A critical step in the development of biosimilars is to demonstrate that there are no clinically significant differences in efficacy and safety compared to the reference product [55]. This involves conducting comparative clinical and preclinical trials, focusing on pharmacokinetics, pharmacodynamics, immunogenicity, and safety parameters [56]. Despite the objective of replicating the therapeutic effects of original biologics, biosimilars are not considered identical—reflecting the inherent complexity and nuances of biologic production [57]. The manufacturing process is where biosimilars differ most notably from their reference biologics [58]. Produced from living cells, biosimilars are sensitive to growth conditions, resulting in potential differences in the structure of the active protein, despite having an unchanged primary amino acid sequence [59]. Biosimilar manufacturers must develop their own cell lines and production processes, and demonstrate that these processes produce a product very similar to the original in quality, safety, and efficacy [12]. Hence, the difficulty regarding the rigor of the standards that accompany these products. However, biosimilars offer important advantages in terms of profitability, since they provide lower-cost alternatives to expensive biological therapies and, mainly, they no longer pay royalties on patents [60].

### 3.1. Patient and Physician Perspectives, Nocebo Effect, and Clinical Outcomes

When addressing patient opinions, it is very important to raise the “nocebo” effect, which is characterized by certain negative reactions from patients to the laboratories responsible for researching and developing new drugs. Also, it emphasizes the negative outcomes of active treatments in clinical settings, such as new or worsening symptoms and adverse events, which are driven by patients’ negative expectations and reactions, rather than by the pharmacological effects of the treatment itself. These are markedly negative perceptions [61,62]. Numerous factors can generate this scenario, such as patient personality, psychosocial and neurobiological effects, poor communication between patient and doctor, and also environmental and cultural influences, which can affect and reduce patients’ adherence to the chosen treatment [63]. A lack of knowledge or adequate understanding of the patient about the true effects of biosimilar-based treatment can generate rejection due to fear. Sometimes, even the treating doctor himself may lack all the necessary information, which can generate negative expectations and nocebo effects, thus reducing the acceptance and clinical and even economic benefits of biosimilars [64].

However, Wu et al. [65] analyzed patients’ perceptions of biosimilars and showed that the majority of them expressed satisfaction with biosimilar treatment. However, the negative perception was reflected in fear of clinical effects and the regulatory approval process. Although most participants understood the potential economic advantages of biosimilars, some incorrectly thought that a lower price was correlated with a lower quality. The same study highlights that a lack of knowledge about biosimilars could be observed in a percentage between 25% and 58%, while up to 51% expressed knowledge about biosimilars. That is, parity can be observed between patients who declared that they were aware of biosimilars and those who did not. On the other hand, information about these medications is generally obtained directly from doctors, pharmacists, and patient associations. Another study on patient opinion shows that among all patients, 66% were unaware of the existence of biosimilars, but the majority were potentially receptive to biosimilar treatment after learning what a biosimilar meant. Despite this, after learning about biosimilars, patients were still concerned about side effects (59%), and long-term safety (50%). Among current users, 43% would switch to a biosimilar and 26% would not (32% were unsure). The patients showed, among other things, interest in learning more about the different benefits and associated final costs [66].

It is essential to provide balanced information on risk–benefit profiles, frame the information to emphasize positive aspects, and encourage shared decision-making and patient empowerment. By increasing their knowledge about biosimilars and being aware of bias-inducing factors, healthcare professionals can help reduce the risk of nocebo effects and improve patient adherence by suggesting biosimilars for autoimmune diseases or treatments against different types of cancer [67]. Adoption of biosimilars in cancer requires critically examining clinical outcomes and patient experiences [65]. Patient organizations, such as the Patient Forum (the WCG Patient Forum is an initiative of WCG (Western Institutional Review Board Copernicus Group), a leading provider of solutions that improve the quality and efficiency of clinical research). The WCG Patient Forum aims to improve the clinical trial process by integrating the patient’s perspective at every stage of drug development and research; the National Patient Advocacy Foundation (NPAF) (a nonprofit organization that represents the patient’s voice in health care policies and practices); and Patient-Centered Drug Development (PFMD) is a global initiative that aims to integrate the patient’s voice throughout the drug development lifecycle. PFMD collaborates with many stakeholders, including patients, patient organizations, industry representatives, and regulators, to promote patient-centered approaches in healthcare [68,69,70,71,72]. Feldman et al. [71] explain that PFMD was created in 2015 with the purpose of uniting people and organizations dedicated to the creation of drugs with the active collaboration of patients, bringing together patients and other groups with relevant knowledge and good ideas about how to effectively involve patients in drug development. Collaboratively, they have developed ’practical guides’ covering the main activities throughout the drug development process. These freely available guides provide practical tips and examples that anyone can use to encourage patient participation. Additionally, these guides help patients better understand the drug development process and how they can participate effectively to ensure that their needs are taken into account. From the perspective of patient organizations, the introduction of biosimilars offers the possibility of more affordable treatment options, thereby increasing access to essential therapies for a broader patient population. However, patient trust in biosimilars may be influenced by their experiences and perceived equivalence to originator biologics [73]. It is crucial to collaborate with patient advocacy groups to collect feedback and address any concerns about biosimilars. In general, patients tend to have the same prejudices about the use of biosimilar medicines, partly due to difficulties in understanding biosimilarity and expressing the need to be well-informed about the change. One of the main causes of the push for biosimilars seems to be, among others, the health budget restrictions of the different countries involved, as well as health insurance. Therefore, how the benefits and drawbacks of both efficacy and safety and the associated costs of these biosimilar medications are communicated to the patient is key [74].

The treating physician plays a vital role in the acceptance and integration of biosimilars into clinical practice, by adequately informing and prescribing such medications [75]. These authors determined that treating physicians play a very important role in the access and availability of information related to biosimilars. The level of familiarity among doctors with biosimilars varied widely: Between 49% and 76% were familiar with biosimilars, while between 2% and 25% did not know what biosimilars were, with variations between different studies. Knowledge measured by the same research was generally lower than self-assessed knowledge. The perceptions of biosimilars also differed: between 54% and 94% were confident in prescribing biosimilars; however, between 65% and 67% were concerned about these medications. There was a tendency to favor originator biologics over biosimilars prescribed, with biosimilars primarily for patients who had not previously received biologic treatments. The main advantages of biosimilars, as perceived by physicians, were cost savings and lower prices compared to biologic originators, while concerns were often related to safety, efficacy, and immunogenicity. Between 64% and 95% of the physicians had negative opinions about pharmacists replacing reference biologics with biosimilars. Physicians’ opinions would be determined by clinical evidence, regulatory guidelines, and their own experiences with biosimilars [76]. For cancer treatments, it is essential to provide oncologists with comprehensive data on the safety and efficacy of biosimilars, as well as real-world evidence from post-marketing surveillance [77]. Educational programs and peer-reviewed and validated publications can help build trust among healthcare professionals, facilitating informed decision-making and adoption of biosimilars in cancer [78].

### 3.2. Impact of the Cost-Saving Potential of Biosimilars on Health Systems

The issue of costs in relation to biosimilars is complex because of many factors that intermingle. The costs of biosimilars can vary widely depending on multiple factors, such as the complexity of the drug, the production infrastructure, the price regulations in different markets, the patient’s situation, and the manufacturer of the product [79]. There are still no conclusive results that allow us to establish standardized long-term costs since they vary according to the factors listed above and will be discussed below. However, some studies have shown that the cost-saving potential of biosimilars is significant for health systems in the United States and Europe. Implementing appropriate strategies can maximize the benefits of these therapies, improving the accessibility and sustainability of cancer treatment. The key is to promote supportive policies, educate healthcare professionals and patients, and invest in continued research and development to ensure the efficiency and effectiveness of biosimilars on the market.

United States

Reduction of costs and financial pressure:

- Biosimilars can significantly reduce treatment costs for healthcare systems in the US due to their lower prices compared to reference biologic drugs [80]. This is crucial in a market with high drug costs, where biosimilars can relieve financial pressure on public health programs such as Medicare and Medicaid.
- The introduction of biosimilars could save the US healthcare system up to USD 100 billion over the next decade, depending on the adoption rate and market competition [81].

Improvement in accessibility and adherence to treatment:

- Reducing biosimilar prices improves accessibility and adherence to oncological treatments, which could translate into better clinical outcomes and a decrease in disease progression [82].
- The availability of biosimilars could expand access to innovative treatments, especially in underserved communities that traditionally have less access to expensive therapies [83].

Europe, meanwhile, significant savings and system sustainability:

- European healthcare systems have achieved significant savings with the introduction of biosimilars, particularly in countries with favorable pricing and reimbursement policies. These savings have been reinvested in the health system to improve the quality of care and access to new therapies [84].
- The adoption of biosimilars in Europe has enabled health systems to maintain financial sustainability in the face of increasing costs of innovative biological medicines [85]

Promotion of competition and price reduction:

- The competition generated by biosimilars has led to a reduction in the prices of original biological medicines on the European market, benefiting both patients and health systems [86].
- The implementation of incentive policies for the adoption of biosimilars in Europe has accelerated competition in the pharmaceutical market, promoting innovation and reducing long-term treatment costs [87]

Strategies to maximize the benefits of biosimilars in the fight against cancer:

Support policies and financial incentives:

- Governments should implement policies that encourage the adoption of biosimilars, such as tax discounts and subsidies for institutions that choose to use these drugs instead of their reference counterparts [88]. These policies can facilitate a faster and more efficient transition toward the use of biosimilars.
- Establishing specific pricing and reimbursement agreements that favor biosimilars is important, ensuring that these are accessible and attractive to both providers and patients [89].

Education and awareness:

- The importance of educational campaigns aimed at health professionals and patients to increase confidence in the safety and effectiveness of biosimilars. This is essential to promote its acceptance and widespread use in cancer treatment [90].
- The need for workshops and continuing education programs for doctors to ensure that they are well [91].

Promotion of research and development:

- Investments in research and development should be encouraged to improve the production technology of biosimilars and optimize their regulatory processes, which can lead to cost reduction and greater availability in the market [92].
- The creation of public–private research consortia to develop new biosimilars, which can accelerate their arrival on the market and increase competition, benefiting patients and health systems with more therapeutic options and lower costs [93].

Continuous evaluation and monitoring:

- The implementation of continuous monitoring and evaluation systems to track the impact of biosimilars on health costs and treatment effectiveness, which can help adjust adoption policies and strategies in real-time [93].
- The creation of open-access databases to share information on the effectiveness and cost of biosimilars, promote transparency, and facilitate informed decision-making in the field of public health [94,95].

### 3.3. The Profitability of Biosimilars against Cancer According to the Perspective of the Actors Involved (Patients, Treating Physicians, Manufacturers)

The cost-effectiveness of biosimilars must be evaluated from different perspectives, both from patients, health systems, and manufacturers. It is important to explore in-depth how these agents impact the health economy in the United States and Europe to become clearer about the general importance of biosimilars in the treatment of critical diseases such as cancer. If we look from the patient’s perspective, we must primarily consider two elements: cost and quality of life.

Reduced costs and accessibility:

- Biosimilars can offer more accessible treatment options due to their lower costs while maintaining the clinical effectiveness necessary to treat serious infections such as the respiratory synaptic virus [96].
- Biosimilars, due to their lower cost, can allow greater patient adherence to treatment, which can translate into better clinical outcomes and quality of life [97].

Impact on quality of life:

- The use of biosimilars can improve patients’ quality of life by making necessary ongoing treatments for chronic conditions—such as cancer—more accessible [98].
- Although some biosimilars may be less effective in certain clinical contexts, their lower costs can compensate for this difference, allowing broader and continued access to treatment [99].

Health system perspective.

Cost Savings for the Health System:

- Biosimilars represent a more economical option for health systems by negotiating lower prices for drugs that are equally effective as their brand-name counterparts [100].

Impact on health policies:

- The adoption of favorable policies toward biosimilars can lead to significant reductions in public health spending without compromising the quality of cancer treatment [101,102].
- The implementation of biosimilar incentives and education programs can accelerate their adoption, resulting in long-term savings for healthcare systems in Europe [103].

Manufacturer perspective.

Impact on competition and prices:

- The competition generated by biosimilars can put downward pressure on the prices of original medicines, promoting a more competitive and accessible market [104].
- Manufacturers can benefit from economies of scale and lower long-term production costs, which allows them to offer more competitive prices without sacrificing profit margins [105].

Challenges and opportunities in the market:

- Biosimilar manufacturers face significant challenges, including high upfront development costs and strict regulations, but they also have the opportunity to capture a considerable share of the global cancer market [53].
- Investment in the research and development of biosimilars is crucial for manufacturers who want to remain competitive and comply with international regulatory standards [92].

## 4. Biosimilar Process and Regulation in Eight Countries on Four Continents

The process and regulation of biosimilars on the four continents are presented through the selection of eight countries represented by the United States and Canada (North America), Australia (Oceania), Japan and South Korea (Southeast Asia), Argentina and Brazil (South America), and finally South Africa (Africa). Each country has its own regulatory challenges for the manufacturing, approval, market dynamics, and growth and innovation opportunities of biosimilars [106]. At the continental level, in South America and Africa, whose economies are developing, the biosimilar market has a comparative and competitive advantage based on economic and social needs, which generates enormous expectations of the rapid growth of these drugs [12]. This is partly due to the need for affordable healthcare solutions as the number of chronic diseases increases in the target population. In South America, Argentina and Brazil lead in terms of regulatory and investment frameworks for the manufacture of biosimilars at the local level [32]. In Africa, South Africa is the most advanced country in biosimilar regulation [30]. However, there are great challenges due to inadequate, dissimilar, and inconsistent institutional frameworks, which prevent the harmonization of biosimilar regulations in different Latin American and African countries as a whole [56]. In Southeast Asian countries (such as Japan and South Korea), North American countries (Canada and the United States), as well as in Oceania (Australia), the respective biosimilar markets are evolving in a more dynamic context based on improved institutional quality and long-term regulatory support for the growing demand for cost-effective healthcare solutions [5,107,108,109]. However, in the United States, there are some barriers on the part of the FDA to approve biosimilars from the moment a patent expires [110,111]. It is important to clarify that each country’s approach to biosimilar regulation reflects its healthcare landscape and public health policy priorities.

### 4.1. Regulatory Guidelines and Approvals for Each Selected Country by Continent

Each country involved in this research requires rigorous data to demonstrate biosimilarity to the original and places a strong emphasis on post-market surveillance, but they differ in their approaches to exclusivity periods, naming conventions, and substitution policies [24,112]. These differences reflect each country’s own health systems and regulatory priorities, balancing the drive for pharmaceutical innovation and the development of a competitive market to improve accessibility to this type of medicine [25]. There is no universal regulatory system, but it varies from one country to another, where some have similarities to others in their regulations and applications, which is why it is interesting to specifically observe some of these countries by continent.

#### 4.1.1. North American Continent (United States and Canada)

In the United States, in the period 2014–2018, there was an unprecedented 50% increase in spending attributed to specialty drugs of biological origin, reaching USD 125 billion. Biosimilars could drastically lower these costs, therefore lowering the quality of therapies, but despite this argument, biosimilars have not yet achieved a boom in this country [113]. The low success in the adoption of biosimilars in the US market would not be due specifically to these drugs, but to differences in the regulatory, legislative, legal, and clinical frameworks, as well as to the payment models used in this country [114]. Despite what is described above, the regulatory framework for biosimilars in the US is designed to try to balance the dual objectives of stimulating innovation in the development of new biological medicines and facilitating the entry into the market of more affordable biosimilar alternatives. Producing a new reference biological drug begins with FDA approval through a Biologics License Application (BLA) [115]. A central element of this regulation has to do with the provision of a 12-year exclusivity period after the approval of the BLA where no biosimilar product can enter the market [116]. This would simply mean prolongation of the camouflaged exclusivity property right, probably under pressure from large pharmaceutical laboratories. However, a biosimilar application can be submitted four years after approval of the reference product. This exclusivity, granted by the FDA, is intended to reward innovation by protecting the reference product, which is different from the original patent protection that the original biological product had [92]. Canada’s approach to regulating biosimilars, known as late-entry biologics (SEB), is designed to foster a balance between encouraging innovation of originator biologic drugs and allowing the introduction of more affordable biosimilar options [117]. In Canada, biosimilars are subject to a rigorous approval process by Health Canada, which involves a comprehensive evaluation of quality, safety, and efficacy compared to the reference biological product [112,117]. On 18 December, 2023, Health Canada announced the creation of the Canadian Medicines Agency. This new entity will provide crucial guidance and organization to promote resilience and future readiness of the Canadian pharmaceutical system, including biosimilars. A notable aspect of the Canadian regulatory framework for biosimilars is that it is similar in principle to that of the United States, although in this case, the protection term of this regulation has to do with the provision of an eight-year exclusivity period for the biological drug original [118]. This period prevents biosimilar manufacturers from using data for their own approval applications, thus protecting the biosimilar manufacturer’s R&D investments and the innovation of the original medicine [119].

#### 4.1.2. Southeast Asia Continent (Japan and South Korea)

In Japan, biosimilar regulation is a key component of the healthcare system, which aims to balance the promotion of innovative biological medicines with the introduction of cost-effective biosimilar alternatives. Japan’s framework, governed by the Pharmaceuticals and Medical Devices Agency (PMDA), requires biosimilars to demonstrate high similarity in quality, safety, and efficacy to their reference biologics [120]. Unlike the United States and Canada, Japan does not have a fixed exclusivity period for original biologics once the patent expires. Decisions are made on a case-by-case basis, allowing flexibility to enter the biosimilar market [121]. The naming convention for biosimilars in Japan follows the International Nonproprietary Names (INN) system, facilitating global consistency [11]. South Korea’s regulatory framework for biosimilars is also designed to ensure the availability of affordable biologic therapies. The biosimilar approval process, overseen by the Ministry of Food and Drug Safety (MFDS), requires extensive comparative studies to establish biosimilarity in terms of efficacy, safety, and quality [25]. South Korea does not specify a different exclusivity period for original biologics, but instead aligns with patent laws for reference products [122]. This approach facilitates a competitive market environment after patent expiration [123]. The naming and labeling of biosimilars in South Korea is designed to ensure clarity and avoid confusion, with specific distinctions from reference biologics. Substitution policies allow the interchangeability of biosimilars at the discretion of the prescribing healthcare professional [118].

#### 4.1.3. Oceania Continent (Australia)

Australia’s regulatory approach to biosimilars is overseen by the Therapeutic Goods Administration (TGA) and is strategically formulated to encourage innovation in biologic drug development while facilitating the entry of affordable biosimilar alternatives [124]. The framework requires biosimilars to demonstrate high similarity to their reference biological products in terms of safety, efficacy, and quality. A feature of the Australian system is the provision of a 5-year exclusivity period for biosimilars, which is shorter than that of the United States and Canada but harmonizes the intention to balance market protection for originator biological products with timely entry into the biosimilar market [26]. In terms of naming conventions, Australia follows a case-by-case assessment, ensuring clear differentiation and security [125]. The decision to switch from a reference biologic to a biosimilar rests with the prescribing healthcare professional, highlighting the emphasis on clinical judgment.

#### 4.1.4. Latin American Continent (Argentina and Brazil)

In Argentina, the regulatory framework for biosimilars is the responsibility of the National Administration of Medicines, Foods, and Medical Devices (ANMAT). It is designed to encourage the development of innovative biological medicines and ensure the availability of biosimilars as affordable alternatives [12]. Regulatory support requires that biosimilars demonstrate similarity to reference products in quality, safety, and efficacy. Argentina does not specify a single period of exclusivity for biosimilars but rather adheres to general pharmaceutical patent laws, which influence the timing of market entry for biosimilars [24]. Regulations require a distinctive name for biosimilars, ensuring clear identification and minimizing potential confusion between the two. Substitution at the pharmacy level is not automatically allowed but requires prescription authorization [126]. Brazil’s approach to regulating biosimilars, overseen by the National Health Surveillance Agency (ANVISA), aims to balance the promotion of innovation in biological medicines with the introduction of cost-effective biosimilar alternatives [12]. Brazilian regulations require biosimilars to provide extensive analytical, nonclinical, and clinical data to establish biosimilarity with reference biologics [127]. Unlike other countries, Brazil, like Argentina, also does not have a specific exclusivity period for biosimilars, and the framework is governed by general patent and data protection laws [128]. Distinctive naming is required for biosimilars for clear identification, and labels must indicate the biosimilar nature of the product [127]. In Brazil, substitution at the pharmacy level is not allowed, being the decision of the health professional [128].

#### 4.1.5. African Continent (South Africa)

Africa is a large continent with 54 states and each country has its own biosimilar regulatory body [129,130]. Some African countries are moving towards adopting WHO, EMA, and FDA recommendations on biosimilarity, quality, efficacy, and safety standards. The regulatory framework for African countries is still in a nascent stage, except in South Africa, Egypt, and Tunisia, which are a little more advanced [30]. On this continent, the commercialization of biosimilars occurs mainly with companies in low-cost regions of China and India that provide copies of biologics [130,131]. The adoption by the African Union of a treaty in May 2018 to establish the African Medicines Agency is an important step towards a harmonized regulatory framework in Africa, which could improve the development and accessibility of biosimilars [132]. These developments highlight Africa’s growing share of the global biosimilar market, with South Africa leading regulatory advances and other countries gradually moving toward establishing their frameworks [133]. The potential for biosimilars in Africa is substantial, considering the size of the population and the need for cost-effective treatment options. South Africa has been at the forefront of biosimilar registrations in Africa and South Africa’s regulatory framework is under the auspices of the South African Health Products Regulatory Authority (SAHPRA) [30]. This country approved its first biosimilars, Filgrastim Teva in 2018 and trastuzumab from Biocon and Mylan Ogivri in 2019 [27]. South Africa established a clear regulatory framework for the approval of biosimilars, and its Medicines Control Council (MCC) published guidelines in 2012 and amended them in 2014 [34]. The estos guidelines align with the criteria of the European Medicines Agency (EMA) and the World Health Organization (QUIEN). These guidelines align with the criteria of the European Medicines Agency (EMA) and the World Health Organization (WHO).

Table 1 shows the number of biosimilars approved in 2023 by each of the eight countries analyzed, where Canada and Brazil lead with 53 and 52 biosimilars approved, then the United States and Australia with 45 and 43 biosimilars. Japan follows with 32 biosimilars, followed by South Korea with 25, Argentina with 24, and finally South Africa with 5 approved biosimilars.

**Figure 2:**
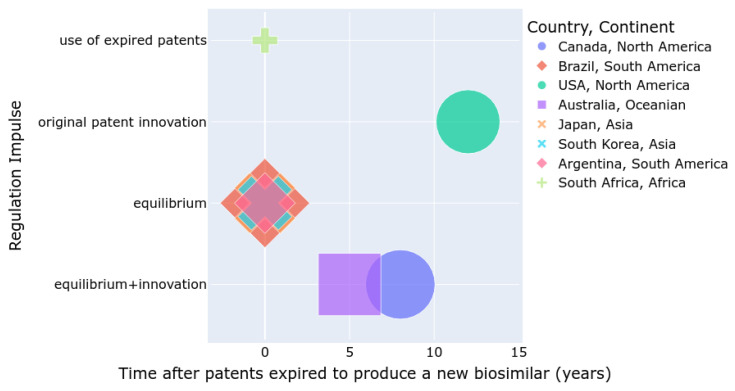
Summary of regulations by country and continent.

Figure 2 summarizes the number of approved biosimilars (size), the time required to develop a biosimilar after a patent expires, and the regulatory incentives for either the innovation of new patented drugs or the use of expired patents for biosimilar production. Countries such as the USA, Canada, and Australia are more inclined to promote innovation, whereas Asian and South American countries tend to balance innovation with the development of biosimilars. In South Africa, the use of biosimilars is increasing, but the number of approved biosimilars is lower compared to other regions.

## 5. Artificial Intelligence Applied to R&D Processes in Biosimilars

The integration of smart technologies to assist in the research and development of biosimilar drugs marks a revolutionary change in the biopharmaceutical industry [12,140,141,142]. AI in healthcare represents a broad spectrum of technologies and methodologies, each playing a critical role in various subdomains [37,143]. When considering the acceleration of new medical applications, particularly in the development of biosimilars, AI and machine learning algorithms are instrumental in improving the efficiency and accuracy of biosimilar drug development processes [144]. IoT plays a vital role in monitoring and managing the biosimilar production method by integrating sensors and connected devices throughout the manufacturing environment, ensuring real-time data collection and analysis [145]. This leads to a more controlled and consistent production process, which is vital to maintaining the quality and effectiveness of biosimilars. These technologies can analyze large amounts of biological data at unprecedented speed, helping to identify potential biosimilar candidates and optimizing their development [36]. They are particularly adept at understanding complex biological systems, which is crucial for creating biosimilars that closely mimic their reference biological drugs. Modeling performed “in silico” in collaboration with mathematics carried out on a reference product to predict its behavior could be an area of potential growth in the application of AI for the development of biosimilars [146]. These methods aim to imitate the behavior of a biological system through a computer program, where the critical model behaves with a pattern similar to that of the real system [36,142,147]. AI is finding an interesting niche in the validation of biosimilars, but this is an area of potential growth [148]. Since the development of biosimilars depends on the demonstration of high similarity to an existing and authorized biological product, regulatory agencies require that the laboratory responsible for carrying out the production of a biosimilar have a comprehensive understanding of the structural and functional characteristics of the reference biosimilar product [149].

### 5.1. Benefits of Artificial Intelligence in the Development of Biosimilars

The use of AI can accelerate the identification of predictive biomarkers to evaluate clinical outcomes [150]. Data derived from the identification of new biomarkers of clinical efficacy can improve the design of clinical trials and, subsequently, result in faster and more efficient tests [151]. Although not solely related to AI, identifying predictive biomarkers and designing more efficient trials, is a key step in the successful development of a biosimilar as described by the FDA [152]. In addition to an analysis of historical data to inform trial design, AI can optimize the trial process itself [153]. By using AI to compare and contrast historical data about the drug and its target, a virtual model could be created that allows simulating the effects of the biosimilar in a low-risk, high-reward scenario in record time compared to what has been done [154]. In this sense, it is interesting to observe in Table 2 the description of the different AI applications used in the development of biosimilars.

Big data analysis further complements the use of these smart technologies by providing deep insights into large data sets related to biosimilars and their parent biological molecules [142]. In this sense, it collaborates in predictive modeling, helping researchers anticipate challenges and opportunities in the biosimilar development life cycle in record time [155]. By analyzing trends and patterns in biological data, ‘big data’ can guide decision-making, from the early stages of drug design to final production [36]. The combination of these smart technologies not only streamlines the various development processes but also improves the precision of biosimilar production. This increase in precision is crucial, given the complex nature of biologics and the need for biosimilars to closely resemble their reference products in terms of safety, efficacy, and quality [149]. By applying these AI methodologies to the development of biosimilars, stakeholders can significantly accelerate the process from laboratory to market, ensuring that these critical medicines are developed efficiently, safely, and at a lower cost [36]. Each AI technology brings a unique set of tools that can address the specific challenges of reproducing biologics, understanding their behavior in biological systems, and ensuring their efficacy and safety in patients.

**Table 2:** Integration of AI and smart technologies in biosimilar R&D.

Application of AI in Biosimilar Development	Description
Machine learning in healthcare for biosimilars [142]	Predictive modeling for biosimilars: critical component used to predict biological and clinical outcomes of biosimilars. Analyzes complex datasets from bioprocessing to clinical trials, predicting how changes in biosimilar production could affect efficacy and safety. Adaptation of biosimilar development: Recognizes patterns in the data to adapt biosimilar development processes, optimize manufacturing conditions, ensure quality, and predict patient responses to biosimilars based on genetic and environmental factors.
Support vector machine for classification tasks [156]	Classification of biological patterns: The support vector machine is used to classify crucial complex biological data to understand the variations between biosimilars and original biological products—including the prediction of the protein structure, functional annotation, or determination of immunogenicity risk of different biosimilar candidates.
Artificial neural networks for biosimilar development [36]	Prediction of complex outcomes: Neural networks analyze vast and complex biological datasets, predicting crucial outcomes for biosimilar development. It is used to understand the relationship between the structure of biological molecules and their function or efficacy, which is fundamental to the replication and validation of biosimilar products. Image analysis in biosimilar research: Artificial neural networks analyze electron microscopy or other imaging techniques to evaluate the quality and similarity of biosimilars.
Deep learning applications for advanced biosimilar analysis [37]	Identification of molecular patterns: Deep learning uncovers intricate patterns in molecular data that are not evident with traditional analysis methods. It is particularly useful to ensure the biosimilarity of complex molecules, leading to more effective and safer biosimilars. Enhancement of radiomics for biosimilar assays: Deep learning can improve image data analysis in clinical trials, helping researchers understand how biosimilars affect disease progression and response, particularly in oncology.

### 5.2. Accelerated Discovery and Development Process

A key step towards an AI-assisted biosimilar development process is to increase the pace and quality of development [157]. In this context, AI is not only a tool to analyze preexisting data but also aims to become a key player in the planning stage of experimental protocols and interpretation of a quantity of data never before treated for the same product under development [158]. The idea is to use it to predict the molecular and physical properties of the original products and quickly and more economically select the molecule most similar to the original [159]. This can be achieved by using machine learning algorithms to indirectly relate product properties to intended clinical safety and efficacy data [160]. In this sense, “in silico” (computational) AI-based molecule design methods seek to maximize the efficiency and effectiveness of the event method at each stage by accelerating the prediction of activities [161]. This covers a very wide variety of methods, but essentially AI would learn a model to predict the relationship between the molecule and the action and would use this model to suggest alterations of the molecules and their expected results in record time [162]. The most immediate biological consequence of this field of computational AI is the use of machine learning algorithms in the analysis of complex biological systems [163]. These algorithms are being implemented to perform what would be considered “big data” analysis or meta-analysis of different data sets, ranging from these ‘in silico’ models to raw data obtained from previous molecular or cellular studies [164]. Molecular modeling methods are commonly used to predict the behavior of a molecule in question or the best way to create a desired molecule [165,166]. This may involve simple 2D structure–activity relationship (SAR) analysis that compares the biological activity of a molecule with its chemical structure or more complex methods that use molecular dynamics or simulations to visualize the behavior of a molecule in atomic detail [167]. These methods are used to identify the most effective changes in an active molecule or the best way to create a replacement [168].

### 5.3. Improved Prediction of Biological Activity

In the development of biosimilars, a detailed understanding of the pharmacokinetics and pharmacodynamics (PK/PD) of the originator product is essential, as this understanding guides subsequent in vitro and in vivo testing that demonstrates biosimilarity [169]. However, this information is not always published, often for confidentiality reasons, and when it is published, it may not be detailed enough [170]. AI can help close the gap by predicting the PK/PD of the original product from the molecular and structural information provided in the reference material [171]. Artificial intelligence with machine learning has proven to be effective in predicting the biological activity of drug molecules, in particular by comparing the molecular interaction of active molecules with their biological target of interest with the physical and chemical properties of the same molecules [172]. This “in silico” approach can help identify molecules that are more likely to have the desired therapeutic effect and also provide an indication of those that are likely to have undesirable effects [173]. Evidence collected by artificial intelligence tools can serve to inform evaluators about the relative safety and possible public health impact of accepting or rejecting a new biosimilar product compared to the original therapy [174]. To look specifically at AI-assisted comparison methods, the identification of potential candidates for the ‘in silico’ routine is, as has been observed, crucial [175]. A routine that compares each biosimilar attribute with the reference product to determine areas of similarity and difference and ranks them in order of potential impact on the efficacy of the biosimilar [176]. In some cases, this may involve developing a mathematical model of the system that can be validated by comparing clinical trial data of the reference product with clinical trial simulations [147]. Stepwise analysis of the type suggested by the EMA involves starting with a comparison of quality attributes and conducting additional comparisons only of those attributes that show disparity [177]. With the development of biosimilars, a thorough analytical comparison between the new product and the reference product should be the basis of a stepwise approach that demonstrates biosimilarity [37]. According to the guidelines of the International Conference on Harmonization, this involves a risk-based approach in which the type and extent of comparisons depend on the complexity of the molecule and any prior knowledge of the critical quality attributes that affect safety and effectiveness [178]. Given that the biosimilar will only be approved if it can be determined that there is no increased risk in terms of safety and efficacy variability, the main objective of any analysis at any stage of development is to detect differences between the biosimilar and the reference product and support that are not due to random variability [179].

### 5.4. Identification of Critical Quality Attribute

An ideal scenario for the development of biosimilars would be to generate an automatic algorithm that takes information about the molecule or the process of change and produces a prediction of the probability of success in demonstrating comparability and the areas that will have the greatest impact from the required studies. This would help in the efficient design of a development program and specific studies for individual products or processes [180]. The approach of the ICH Q8 (R2) guideline on pharmaceutical development is based on the design of a quality product and its manufacturing process by evaluating the impact of raw materials and process parameters that can affect the quality profile of the product [181]. This offers a decision tree methodology to establish a specific design space to ensure the quality of a product [182]. Therefore, quality is ensured if the product is manufactured within the design space; the set of predefined conditions is established to ensure the quality of the process and the product [183]. This methodology is applied by generating data from studies designed to reflect the probability of analytical success in detecting a change in a critical quality attribute [184].

### 5.5. Machine Learning and Deep Learning Integration

One application of machine learning that is poised to have a major impact on the development of biosimilars is the prediction of the higher-order structure (HOS) of proteins and the evaluation of comparability with innovative drugs [185]. Currently, the development of a biosimilar involves extensive use of analytical methods to evaluate the comparability of the biosimilar with the innovator drug [149]. Machine learning algorithms can be trained with data from synchrotron radiation circular dichroism (SRCD) experiments [186] and hydrogen/deuterium exchange mass spectrometry (H/DX-MS) that are used to evaluate the HOS protein, to create predictive models of the HOS protein from primary data and secondary protein sequence data [187]. These predictive models can enable rapid assessment of the comparability of a biosimilar with an innovator drug without the need to repeat costly and time-consuming HOS experiments. Artificial neural networks have been used to find patterns in gene expression data in an attempt to determine correlations with protein abundance and function [188]. These applications corroborate that machine learning has already proven to be valuable in the more traditional biochemical and biological field of biosimilar development.

### 5.6. Application of AI in Pharmacovigilance

Simulation studies have shown a high probability of detecting rare adverse events with AI methods compared to traditional statistical methods that use much smaller sample sizes [189]. This is important because it reduces the amount of exposure a patient has to a medication before an adverse event is detected. The adverse events with the biosimilar can then be compared with those of the reference product using unsupervised machine learning methods to assess whether there has been a significant difference [142]. This type of analysis is described as a “probabilistic” comparison and is more powerful than traditional statistical methods because it identifies and evaluates changes in the probability of events rather than specific events and associated tests [190,191]. On the other hand, AI methods can help detect adverse events by allowing the comparison of structured and unstructured data patterns of patients treated with a biosimilar with the data of those treated with the reference product [156]. This is achieved through data mining techniques, which extract information about adverse events and establish whether there is a causal link between the event and the drug through an inference system on event terms and drug definitions [192,193].

### 5.7. Collaborative AI Platforms for the Development of Biosimilars

Through increased use of machine learning technology and predictive analytics, collaborative AI platforms are being developed for reporting and analysis of adverse events of biosimilars globally [194,195]. Currently, adverse event data are collected in different parts of the world and stored in very powerful databases, and systems for reporting adverse events and software for cause-and-effect analysis vary between organizations and countries, making this still a complex process [196,197]. EudraVigilance has created a system for simplified reporting of individual case safety reports (ICSRs) using a structured electronic format and input mask [198]. However, a significant proportion of adverse events remain unreported. The aim of the AI platform would be to provide global access to stored adverse event data using a common data model and automated multilingual causality analysis to standardize the process [199,200].

### 5.8. Natural Language Processing in Biosimilar Development

The integration of NLP into medical studies, particularly through the use of advanced pre-trained models and optimization techniques, is increasingly applied in the development of biosimilars. There are models that adapt the use of NLP by training on large datasets to find connections between data, such as sentences and words. In the case of biosimilars, these models have been used to identify chemical relationships between genes and sequences that can generate diverse structures that capture numerous dependencies, markers, and diseases based solely on medical documents and new candidate molecules for a novel design [201,202,203]. Additionally, natural language processing quickly analyzes large amounts of scientific literature and regulatory documents to aid biosimilar development. It extracts information, trends, and relevant data points to inform development strategies and regulatory considerations [204].

These applications demonstrate how NLP (as shown in Table A1 as Appendix A), particularly transformer-based, language- and multimodal models, is revolutionizing the biosimilar landscape. Using the power of NLP, researchers and developers can accelerate the development process, improve regulatory compliance, and ultimately bring safer and more effective biosimilars to market. Models such as M-FLAG: medical vision–language pre-training with frozen language models and latent space geometry optimization using advanced vision–language pre-training techniques with frozen language models and latent space geometry optimization [205]. These models facilitate the integration and analysis of complex data from various modalities to identify new biological relationships and drug candidates. The frozen language model helps to learn zero-shot electrocardiograms (ECG), demonstrating how frozen language models can be applied to zero-shot electrocardiogram learning, speeding up the identification of relevant patterns in clinical data, thus supporting biosimilar evaluation and monitoring in clinical studies [206]. Med-UniC: unifying cross-lingual medical vision language pre-training by reducing bias focuses on unifying medical vision language pre-training across languages by reducing biases, improving the accuracy and generalization of NLP models in extracting and analyzing multilingual medical data, crucial for biosimilar development in a global context [207]. These advances contribute significantly to the efficiency and effectiveness of the development and utilization of biosimilars.

## 6. Application of Biosimilars in Cancer

The biopharmaceutical industry continues to research new drugs to treat a wide range of oncological diseases. As technology advances, these drugs are becoming more efficient and more precise; however, at the same time and compared to other classes of therapies, the costs patients must face for cancer treatments are unattainable for most budgets [59]. In an attempt to address the health and economic consequences of these high costs, the entry of biosimilars into the global healthcare system has facilitated the possibility of more affordable biologic therapies entering the healthcare space. However, its roots in oncological clinical practice have shown a delay compared to its success in other areas such as the treatment of autoimmune, inflammation, and cardiovascular diseases [208]. Reasons may include limited clinical evidence and short-term follow-up data, and general preference controls for the original due to its high perceived clinical value and superior efficacy [209]. There may also be a factor of inertia, in the sense that medications historically prescribed for therapies continue to be so due to a trust process between doctors and patients.

### 6.1. Modeling Behaviors of Active Compounds

The superiority of the study of cell states focused on proteins—and their mutual interactions—is that masses of cancer cells are always measurable and then—in the context of a mass of normal cells—allow for an early diagnosis of cancer [210]. The second category of ODE biological systems consists of those in which the dynamics of these proteins or some of the subcellular machinery are followed over time [211]. There are also biological elements that affect the behavior of anticancer drugs, which should be incorporated into the model. These include the age, weight, and sex of the patients (based on pharmacokinetic data), but also the other cytostatic or cytotoxic drugs in combination therapies and the patient’s immune system, which would be important when immunotherapy is combined with other types of cancer [212]. Another modeling tool is the system of ordinary differential equations (ODE), which is essential for investigating and modeling the behavior of active compounds [213]. These active chemicals include anticancer drugs, small molecules, which are often considered cytostatic or cytotoxic agents, interleukins, cytokines, antibodies, etc. The advantage is that ODE techniques allow the use of data obtained from early and late clinical stages, but also from preclinical in vivo and in vitro experiments [214]. All of these types of data can be used to decide on effective administration schedules. Two elementary classes of EDO systems have been very popular in research [215]. The first class is called the type of time-dependent constant coefficient type, which only includes a linear structure of drug pharmacokinetic data. These types of systems are used when anticancer compounds have only renal elimination or few metabolic steps. For more complex profiles or when the drug is made up of peptides or monoclonal antibodies, the drugs are those of the second family, systems of periodic coefficients in time, with time-dependent kinetics of the drug in the system [216].

### 6.2. Spectroscopy Data Analysis

Anatune and Chemometrics detailed guidance should be documented [217]. While the guide does not establish the minimum number of samples necessary for a repeatable measurement, it does clarify that individual spectra from a series of completed analyses should be integrated into a single spectrum for better repeatability that addresses possible variations in laser energy, differences in the point of focus, and small variations in the alignment of a sample with the microscope objective. Currently, available guidelines for study measurements of biosimilar versions generally do not discuss this step. Therefore, the normalized spectrum of the untreated sample should be inspected in detail. If both the sequential measurement lines and the spectra are at multiple points at approximately the same distance due to the influence of temperature changes on the laser excitation and differences in the zero point, the maximum position of the LSCB band should be within 1 cm^−1^, in some devices, a displacement of at least up to 4 cm^−1^ could still be eligible. If changing amplitudes are common and consistent with sequential measurements (most signatures are identified in Raman measurements over a decade), it is assumed that the samples are treated appropriately. The monoclonal antibody rituximab, for example, is licensed for use in rheumatoid arthritis and non-Hodgkin lymphoma (NHL), where the price drop for these indications can be up to 30 times the original [218,219]. The existence of a biosimilar rituximab would generate considerable savings and would probably expand the authorized indications. This may increase access for patients with conditions in which its use is currently uneconomical. In contrast, the use of erythropoietin-stimulating agents for the treatment of anemia secondary to cancer has been controversial due to cost, safety, and concerns about potential adverse effects on cancer progression [220,221]. Monoclonal antibodies (mAbs) are an important component of targeted therapy and are widely used in the treatment of many types of cancer [222]. Among the various classes of biologics, tumor necrosis factor (TNF) antagonists and erythropoietin stimulating agents (ESAs) emerge as the leading contenders in terms of sales and clinical experience [223]. Based on the availability of match data between original biological products and biosimilars, AI has the potential to more accurately predict the efficacy and safety of biosimilars in record time and with greater certainty [35,36]. The use of the National Comprehensive Cancer Network (NCCN) Oncology Drug Compendium, composed of evidence-based data, to compare biosimilars with their originators in mAbs and other biologics has been an important advance [224]. Biosimilar medicines supported by FDA and EMA approval for cancer treatment offer a variety of tested and approved options to combat cancer [54,225].

**Figure 3:**
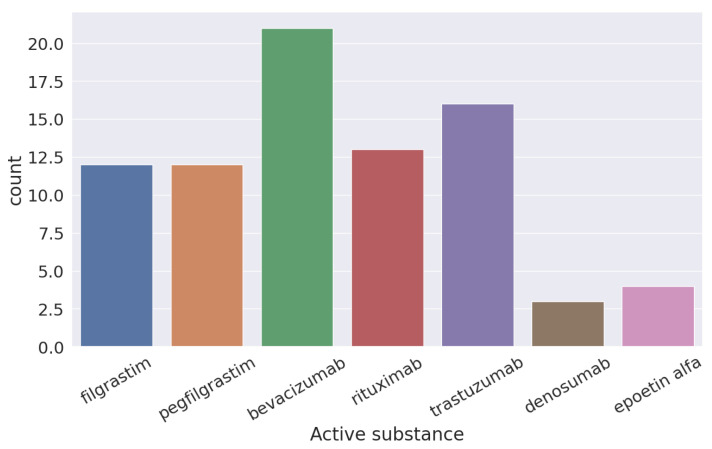
Active components approved by the FDA for use of cancer treatment from “bevacizumab”.

In Figure 3, you can see the six different active components used for the treatment of cancer according to the FDA of the original molecule of bevacizumab.

**Figure 4:**
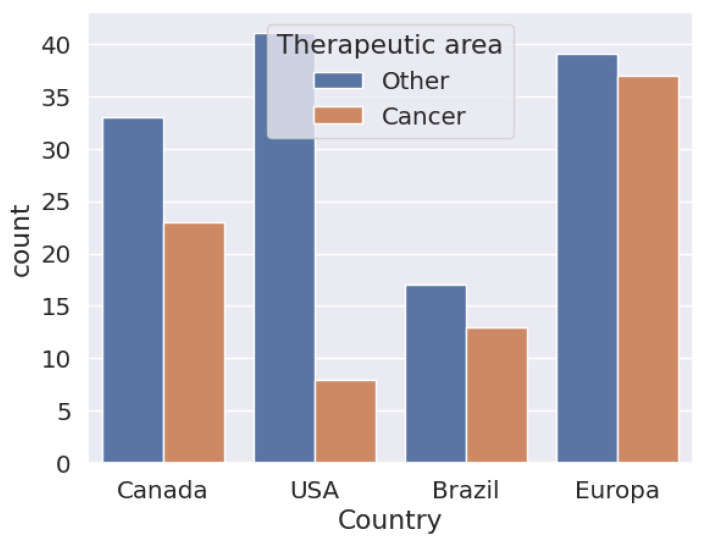
Biosimilars approved in Canada, the United States, Brazil, and Europe to be applied in cancer treatments.

In Figure 4, one can see the biosimilars approved until 18 April 2024. It can be seen that in the European Union, the number of biosimilar approvals used for cancer treatment is similar to those used for the treatment of other diseases, compared to the United States, where the approval of biosimilars for cancer is less than 50% in relation to the use of biosimilars for other diseases. In Brazil and Canada, the proportion of the distance between them is not so marked. In all cases, biosimilars for the treatment of cancers than for those that are other than cancer. However, in proportion to the number of diseases treated by biosimilars, cancer has been well studied and has a higher proportional approval.

**Figure 5:**
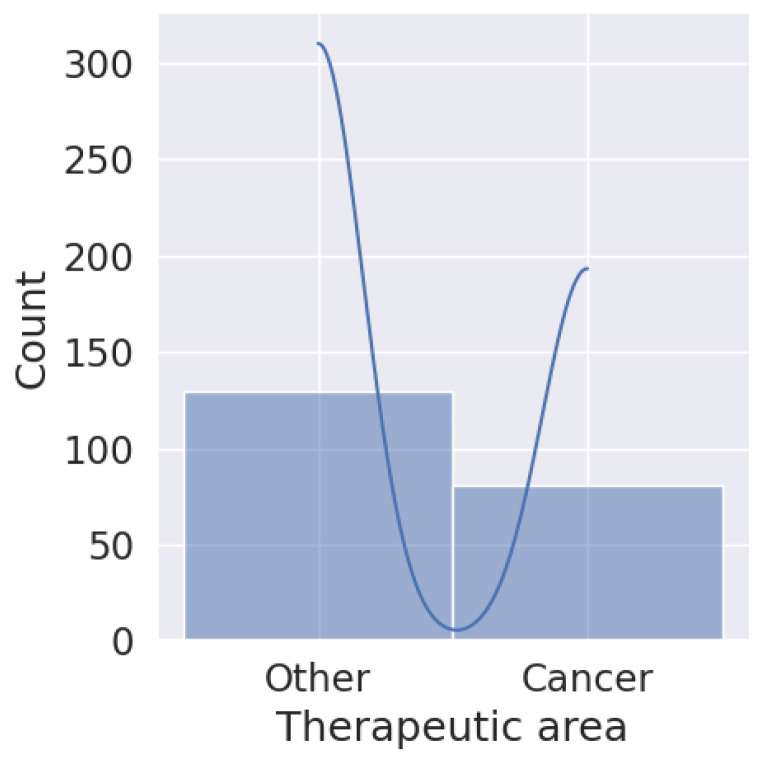
Total biosimilars approved for cancer treatment based on investment.

In Figure 5, it can be seen that the approval of biosimilars for cancer treatment represents 40% of the total approved biosimilars. With the exception of the United States, which does not invest in the massive approval of biosimilar molecules for oncology use, other countries, and more so emerging ones, invest a lot of their studies in the approval of drugs for oncology use.

The potential for significant cost savings in the healthcare system is a key factor in the use of biosimilars in such expensive treatments [226]. Healthcare professionals play a crucial role in integrating these biosimilar-based therapies into daily practice in the fight against cancer [227]. Due to the complexity and heterogeneity of each country’s different clinical, population, and regulatory environments related to cancer treatments, prescribing decisions, and treatment regimens are based on a multitude of different factors and variables [228]. To alleviate this multitude of multiple events, there is an artificial intelligence-based clinical decision support system (CDSS) designed to help cancer researchers make the most optimal and personalized treatment decisions for each patient [229]. CDSS could be used to evaluate the implications of switching to a biosimilar compared to the originator product [230,231]. An AI model that compares the treatment results of biosimilars and their originator products is likely to become an essential tool in determining whether substitution is an appropriate option for cancer patients at both an individual and population level [232]. The development of such a model would require the integration of large-scale clinical data, including patient demographics, disease characteristics, treatment history, and clinical outcomes [233]. Additionally, the AI model should take into account the specific pharmacokinetics and pharmacodynamics of the biosimilar and originator.

## 7. Discussion

The biopharmaceutical sector is experiencing a significant change with the increase in biosimilars, especially after the expiration of multiple patents on biological molecules that have been occurring since the last decade [1,2,3,4]. As we have already seen, biosimilars are complex biological entities that replicate the original biological medicines since they share primary protein sequences with their reference drugs, which is a requirement for their development, but the conditions of the biotechnological process can induce minor variations in the higher-order structures of the resulting proteins [19]. Unlike generic drugs, biosimilars face unique challenges due to differences in the manufacturing process that can affect the structure of the active protein, requiring rigorous regulations to ensure their efficacy and safety [11]. These large molecules are derived from living cells and share primary protein sequences with their reference products. These variations can significantly influence the efficacy and safety of the biosimilar. For example, post-translational modifications such as glycosylation can alter the stability of the protein, its immunogenicity, or its biological activity. This underlines the need for comprehensive and comparative characterization with the reference product, using advanced techniques such as mass spectrometry and liquid chromatography, which are essential to establish biosimilarity [37]. However, the manufacture of biosimilars can introduce minor but significant variations that require rigorous evaluations to confirm that these products maintain the efficacy and safety of their original counterparts [5,37]. Physicochemical characterization plays a crucial role in this process, ensuring structural and functional similarity to the original drug [23]. The manufacture of biosimilars introduces a spectrum of variability not seen in the production of small, non-biological medicines. Therefore, regulatory bodies such as the FDA in the US and the EMA in Europe have established strict criteria for the evaluation of biosimilars. These include the demonstration of similarity in terms of purity, potency, and biological activity through a set of analytical, preclinical, and clinical studies [5]. One of the central debates in the regulation of biosimilars is the level of evidence necessary to demonstrate their comparative safety and efficacy. Although some argue in favor of large clinical trials that replicate those conducted for the original drugs, others advocate a more pragmatic approach that relies on solid analytical characterization and pharmacokinetic studies, reserving clinical trials for situations where analytical analyses suggest significant differences [23]. The key is that the development of biosimilars provides more accessible therapeutic alternatives. However, the complexity of their development and different regulations require a collaborative approach between developers, regulators, healthcare professionals, and patients to ensure that the benefits of biosimilars are fully realized in the context of global and equitable healthcare [15,25]. The regulatory frameworks for biosimilars vary significantly between regions. In North America, the FDA and Health Canada have implemented robust systems that offer post-patent exclusivity periods to protect original biologics while facilitating the entry of biosimilars [116,117]. In contrast, Japan and Brazil have more flexible approaches and do not establish fixed exclusivity periods, allowing greater competition and access to biosimilars [12,121]. This diversity of regulatory approaches reflects the different public health policies and economic needs of each country. An industrialized country with health plans that generate benefits for its members is not the same as a plan intended for developing countries [234], where, except for Brazil and partly Argentina, medications do not reach patients due to the high prices that patients must pay and also the lack of adequacy of medical personnel in prescribing biosimilar medications [12]. The same author cites the fact that the emergence of biosimilars, driven by the numerous expirations of biological products, which address the demand for more affordable public health solutions around the world is increasing every day. These biosimilars, although designed to be similar to their biologic counterparts, are not identical due to inherent variations in their manufacturing, highlighting the critical need for meticulous regulatory evaluations to ensure comparable safety, efficacy, and quality [22]. Different countries around the world seek to competitively regulate regulations to homogenize study and analysis parameters, as well as their approvals [235,236,237]. The growing adoption of biosimilars is not only due to patent expirations. Also due to its potential to reduce costs in the treatment of complex diseases such as autoimmune, ocular, and cardiovascular diseases, and especially those related to oncology [5,209]. Historically, in these treatments, high costs have been associated with royalties and intellectual property protections, making quality state-of-the-art treatments practically unattainable for populations in poorer countries or those with fewer resources [8,93]. Globally, regulation varies considerably, reflecting differences in policy priorities and health systems [18,169,238]. In North America, for example, legislation continues to favor post-patent exclusivity periods protecting original biologics, while in Europe and some regions of Asia there is a trend toward reducing these periods to encourage greater competition and accessibility [239]. Regulatory frameworks in developing countries are still evolving, with challenges including a lack of homogeneity and the need to adapt to guidelines from bodies such as the WHO and EMA [12]. These obstacles could be attributed equally and speculatively to the strength of laboratories that are at the forefront of the development of new molecules to prevent biosimilars from taking away the market in fundamentally novel cancer treatments. In the field of oncology, biosimilars offer significant potential to reduce the costs of treatments against different types of cancer [240,241,242]. However, its integration into clinical practice has been slow, facing challenges such as the need for solid evidence to demonstrate its equivalence with original biologics and the reluctance of health professionals to adopt less expensive alternatives due to concerns about their effectiveness and security [37,208,243,244]. As more cancer biosimilars enter the market, it will be crucial for health systems to adopt policies that not only facilitate their adoption but also ensure post-marketing monitoring to validate their long-term safety and efficacy. Artificial intelligence is transforming the development of biosimilars, from prediction of molecular structures to optimizing manufacturing processes [140,141]. Machine learning and natural language processing facilitate clinical trial design, safety monitoring, and production efficiency [36,143]. These technologies not only promote faster and cheaper development but also improve the accuracy of comparability of biosimilars with reference biologics. One of the most important applications of AI in the development of biosimilars is the prediction of molecular structures [245]. Using machine learning algorithms, researchers can now accurately model complex three-dimensional protein structures that are crucial to the therapeutic efficacy of biosimilars. These models help anticipate how small differences in amino acid sequence or post-translational modifications can influence the function and immunogenicity of the products [140]. The ability to anticipate these characteristics essentially allows developers to optimize cell culture conditions and purification processes to produce biosimilars that are truly comparable to the originator products. Artificial intelligence is also revolutionizing the way biosimilars are manufactured. Through the use of advanced process control systems that integrate machine learning, companies can monitor and adjust production conditions in real-time to ensure product consistency and quality. This not only improves efficiency and reduces waste, but also ensures that each batch of biosimilar meets the strict similarity standards required by regulatory authorities [36]. Machine learning and natural language processing significantly facilitate the design and management of clinical trials for biosimilars. Some advanced algorithms can analyze large volumes of data from previous studies to identify the most relevant parameters that should be evaluated, thus optimizing study designs to be more effective and less expensive. Furthermore, AI can continuously monitor emerging safety data to quickly identify any signs of adverse events, thus improving patient safety during clinical trials [143]. In monitoring the effectiveness of biosimilar therapy, AI has been used to evaluate laboratory biomarkers, showing significant improvements in patients with various parameters in rheumatoid arthritis who receive biosimilar treatment.

Finally, it is important to highlight in this discussion that the current trends and future perspectives for the use of biosimilars in the treatment of cancer are multiple although they depend on numerous internal variables specific to each molecule, but also external ones, dependent on the evolution of the regulations, the availability of patient associations, and treating physicians to accept or not the more massive use of this type of medication. The use of biosimilars in cancer treatment points to increased adoption and promising future prospects. To maximize the benefits of these medicines, healthcare professionals must integrate biosimilars into their daily practice through educational strategies, incorporation of patient associations, incentive policies, and an evidence-based approach. This will not only improve the accessibility and quality of cancer treatments but will also contribute to the sustainability of health systems. If we look at current trends, we could name four main ones:

- Gradual increase in the approval and use of biosimilars: In recent years, a significant increase in the approval and use of biosimilars in cancer treatment has been observed, especially in Europe and the United States. In this sense, the authorization of biosimilars has increased as regulatory agencies such as the EMA and the FDA have developed clearer and more precise regulatory frameworks for their evaluation and approval [225].
- Expansion of competition and cost reduction: The introduction of biosimilars has encouraged competition in the pharmaceutical market for biomolecules, leading to lower prices for both biosimilars and original biological medicines. This competition benefits the different health systems involved in funding programs and the patients by making treatments more accessible [101].
- Adoption in developing countries: Biosimilars are gaining ground in developing countries precisely because of their lower cost compared to reference biologics. The trend is particularly important in resource-limited regions, where biosimilars offer a viable option for the treatment of chronic diseases such as cancer [246].
- Expansion of the treatment portfolio: The number of biosimilars available on the market is expected to continue to increase. In the coming years, biosimilars will be here to stay, and more massive approvals are a matter of time, and therefore a broader range of oncological indications will be approved, thus expanding treatment options against an even greater variety of treatments against different types of cancer [247].

Regarding future prospects, the observed panorama is quite encouraging for a good proportion of beneficiaries. Among them, there are four possibilities.

- Improvements in production technology through the use of more massive AI: Innovations in biosimilar production technology should continue to improve the efficiency and quality of these medicines, with the greater use of different AI tools, which could further reduce costs, improve quality and safety, and obviously, accessibility [248].
- Integration into standard treatment protocols: Biosimilars are likely to become increasingly integrated into standard treatment protocols for various types of cancer, allowing healthcare professionals to offer more accessible, flexible, and cost-effective treatment options. This integration will likely accelerate as more data on the long-term safety and efficacy of biosimilars become available [249].
- More favorable policies and refunds: Policies for biosimilars, both in Europe and other markets, would promote greater adoption of these medicines, making healthcare systems more financially sustainable and lowering payment premiums for all involved [85].
- Greater education and confidence of patients and treating physicians: Education and awareness of the benefits and safety of biosimilars as they become available will be crucial to increasing trust among healthcare professionals and patients, facilitating their adoption and regular use in clinical practice [235].

It is important to highlight some strategies for the effective integration of biosimilars into daily practice. We identified at least six of these strategies.

- Training and continuing education: Regular training on biosimilars is essential for healthcare professionals to fully understand their benefits, limitations, and the regulatory framework that supports them [90,91].
- Development of updated treatment protocols: It is important to generate and develop an update of the treatment guidelines to include specific recommendations on the use of biosimilars, ensuring that professionals have a clear framework for their prescription and monitoring [12].
- Promoting evidence-based adoption: It is critical to building trust between healthcare professionals and patients [250].
- Implementation of financial incentive policies: The implementation of financial incentives for the prescribing of biosimilars could encourage healthcare professionals to opt for these treatments, ensuring that economic benefits are passed on to patients and the health system, in general, [251].
- Monitoring and evaluation of results: The creation of robust monitoring and evaluation systems for patients who use biosimilars, allowing adjustments in health practices and policies over time and as necessary to optimize the effectiveness and efficiency of treatment, since being biomolecules, reactions can vary from patient to patient [252].
- Promotion of participation in clinical studies and information on patient associations: Participation in biosimilar clinical studies can help healthcare professionals stay up-to-date with the latest research improve the adoption of these treatments in their daily practice and inform respective patient associations [95,246].

## 8. Conclusions

Biosimilars represent a transformative development in the biopharmaceutical industry as cost-effective alternatives to patented biologics, marking an important milestone and addressing the critical need for more accessible treatments in both developed and developing countries. The global narrative of biosimilars is rich and varied, characterized by the diversity of regulatory frameworks and the ramified evolution of different markets. This diversity reflects the unique challenges and opportunities that different regions face when integrating biosimilars into their healthcare systems. Developed countries often lead regulatory innovations, while developing countries struggle to establish frameworks that can accommodate these complex biologics. Market developments in regions such as North America, Southeast Asia, South Africa, and South America, along with Australia’s trajectory, highlight a growing recognition of the role biosimilars can play in improving accessibility and healthcare affordability. With the future ahead, the success of the biosimilar industry depends on a harmonized approach to its regulations on a global scale. Such harmonization would not only facilitate smoother entry into the biosimilar market but would also ensure consistent standards of safety and efficacy across borders. Other important challenges lie in the quality of the infrastructure used for research, development, and manufacturing, which in some developing countries is often poor, of low quality, and with inefficient public health policies and terrible governance, as is the case with Brazil and Argentina in South America and with South Africa in Africa, which can make regulatory harmonization of different intra- and inter-border biosimilars difficult. The global landscape for biosimilar regulation is characterized by great diversity, reflecting different national healthcare systems, regulatory priorities, and strategies to balance pharmaceutical innovation with market competitiveness and accessibility. This lack of harmonization can create barriers to entry for these biomolecules in different markets, slowing their adoption and limiting their impact on the affordability of quality care for patients who need it most. Therefore, due to the cultural complexity of the countries involved and their disparate socioeconomic problems, it is important to find a more global understanding of the regulations related to these medications. In essence, its development is a delicate balance between replicating the positive therapeutic effects of the original biologics. Although there is a universal commitment to ensuring the safety, efficacy, and quality of biosimilars, approaches to exclusivity periods, naming conventions, and substitution policies vary significantly from country to country. This diversity reflects the delicate nature of finding the unique balance between fostering pharmaceutical innovations by funding basic research into new molecules and encouraging their intellectual protection through patents, while at the same time fostering a competitive market for pharmaceutical biosimilars to improve access to quality medications for a low-income patient population. Countries such as the United States and Canada emphasize a balance between stimulating innovation through periods of exclusivity and facilitating market entry for biosimilars, while Japan and South Korea offer more flexible approaches to biosimilars. Australia, Argentina, and Brazil also adapt unique frameworks, each tailoring their biosimilar policies to their legal and health systems. South Africa established a clear regulatory framework for the approval of biosimilars through its Medicine Control Board. Despite the growth in specialty medicine spending and the potential of biosimilars to reduce healthcare care costs, the adoption of biosimilars varies significantly between countries, influenced by regulatory, legislative, legal, and clinical frameworks, as well as the delivery payment. In oncology, biosimilars have demonstrated their ability to significantly reduce treatment costs. Biosimilars of agents such as trastuzumab and rituximab are providing more affordable options for patients, without compromising efficacy or safety. The application of AI in the development of biosimilars is revolutionizing this field, facilitating processes from molecule design to production optimization and quality control. Machine learning and deep learning systems can predict protein stability and optimize cell culture conditions to maximize the production of therapeutically relevant biosimilar proteins. Due to the cultural complexity of the countries involved and their disparate economic and social problems, it is important to find a more global understanding of the regulations related to these medications. In essence, biosimilars represent a sophisticated and nuanced class of therapeutic agents in the pharmaceutical landscape. Their development is a delicate balance between replicating the therapeutic effects of original biologics, represented by biosimilars and navigating the complexities of research into new biologics, which involve significant investments and long testing periods throughout the development phases development that can reach 10 years, which considerably increases the prices of these exclusive drugs and makes them unaffordable to ordinary patients, regardless of their nationality of origin.

Among the most significant findings of this research, we can mention the importance of global regulations and challenges; the impact of the use of artificial intelligence in the development of biosimilars; the significance of the adoption of biosimilars for the treatment of different types of cancer; the accessibility and affordability of biosimilars, particularly in developing countries and those with health insurance problems; the collaboration and international support that these molecules can mean for developing countries; the importance of the opinion of patients and doctors for the most efficient communication about the quality of these medicines. Based on each of these findings, different recommendations, actions, and justifications are addressed to improve the global understanding of these important medications, such as biosimilars.

- Global regulations and challenges: The regulatory framework for biosimilars worldwide is disorganized and without unifying criteria, where each country establishes the rules according to its own health needs and interests. Likewise, there is an interest in extremely strict regulations in all countries, most of the time there is a lot of disparity, fully impacting the quality of the medicine and, therefore, the safety of the patients involved, generating fear in their use.
  – Recommendation: Implement a harmonized international regulatory framework to facilitate the approval and adoption of biosimilars in different parts of the world.
    * Actions: Establish a global working group composed of representatives from major regulatory bodies such as the FDA (US), EMA (Europe), PMDA (Japan), patient representative groups, and other authorities responsible for the manufacturing and distribution of medicines. Develop unified guidelines for biosimilar approval processes, including clinical trial requirements, quality standards, and post-marketing surveillance. Create an international database to share regulatory data and best practices.
    * Justification: Currently, regulatory requirements for biosimilars vary significantly between countries, resulting in delays and increased costs for manufacturers, who must navigate multiple regulatory landscapes generating mistrust and uncertainty. A harmonized approach would streamline the approval process, reduce duplication of efforts, and facilitate faster access to biosimilars around the world.
- Use of artificial intelligence in the development of biosimilars: The use of artificial intelligence (AI) in the development of biosimilars for cancer treatments offers both positive and negative contributions. Among the positive contributions, can be said to be the acceleration of development and cost reduction, where AI can analyze large volumes of complex biological and clinical data quickly and efficiently, significantly accelerating the process of discovery and development of biosimilars. Similarly, artificial intelligence improves the efficiency, precision, and personalization of treatments, enabling the identification of new therapeutic targets and the design of molecules that precisely imitate the properties of original biological medicines. It allows a detailed analysis of the genomic and proteomic profiles of patients, helping to predict the behavior of biosimilars in different clinical settings, and optimizing the formulation and dose of these drugs. The potential negative impacts are data dependence and risk of bias since we are highly dependent on the quality and quantity of data available and therefore there is a risk that AI models reproduce or amplify biases existing in the data, which can result in the under-representation of certain patient groups or incorrect decision making. Ethical challenges are also faced regarding the transparency and interpretability of AI models used in decision-making clinics and the development of treatments.
  – Recommendation: Invest in artificial intelligence and machine learning technologies that help improve the efficiency and precision of the biosimilar development process.
    * Actions: Allocate funding for R&D initiatives focused on the application of artificial intelligence in the development of biosimilars. Foster partnerships between pharmaceutical companies and technology companies specializing in AI. Deploy AI-powered platforms to predict molecular structures, optimize cell culture conditions, and conduct virtual clinical trials. Integrate artificial intelligence tools into regulatory review processes to assess biosimilarity and predict clinical outcomes.
    * Justification: Artificial intelligence technologies can analyze large data sets more quickly and accurately than traditional methods, identifying optimal biosimilar candidates and predicting their clinical performance. This can significantly reduce the time and cost involved in bringing biosimilars to market, ensuring that patients receive safe and effective treatments sooner.
- Adoption of biosimilars in cancer treatment: At the molecular level, biosimilars are designed to be highly comparable to the original biologics in terms of structure, function, and biological activity. Through extensive characterization and comparability studies, biosimilars ensure that any molecular differences do not compromise the safety or efficacy of treatment. A strict evaluation of immunogenicity and stability ensures that these drugs can be used safely and effectively in the treatment of cancer, providing a viable and more accessible alternative to original biological medicines.
  – Recommendation: Increase comparative clinical studies and educational programs for patients and health professionals on the safety and efficacy of biosimilars in oncology compared to the reference ones. Focus on points such as structural and functional equivalence, glycosylation and post-translational modifications, the main mechanisms of action, further studies of comparability and immunogenicity, and finally, stability and purity.
    * Actions: Conduct large-scale, multicenter clinical trials that compare biosimilars with their reference biologics in cancer treatment. Develop comprehensive educational modules and certification programs for oncologists and other healthcare providers. Host international conferences and seminars to share trial results and real-world data on the efficacy and safety of biosimilars. Generate and communicate relevant information to patient associations to improve understanding of biosimilars. Collaborate with medical societies to update clinical guidelines that incorporate biosimilars.
    * Justification: Despite their potential, biosimilars have faced slow adoption in cancer due to more limited clinical evidence relative to reference molecules, and there are concerns about their efficacy and safety. Robust comparative studies and continuing education could build greater confidence among healthcare providers, leading to greater acceptance and use of biosimilars in cancer treatment.
- Accessibility and affordability (cost) of biosimilars: Biosimilars have had a transformative impact on cancer treatment in both the United States and Europe, providing significant benefits in terms of reducing costs, expanding access to treatments, and fostering competition and innovation in the pharmaceutical market. These benefits are essential to improve the sustainability and effectiveness of health systems, allowing better resource management and broader, more affordable care for cancer patients.
  – Recommendation: Establish pricing and reimbursement policies that encourage the adoption of biosimilars, especially in developing countries.
    * Actions: Implement government subsidies and financial incentives for biosimilar manufacturers to reduce production costs. Negotiate wholesale purchasing agreements with manufacturers to ensure lower prices for national healthcare systems. Develop reimbursement policies that favor the use of cost-effective biosimilars over more expensive reference biologics. Provide grants or low-interest loans to local companies in developing countries to develop biosimilar manufacturing capabilities.
    * Rationale: High treatment costs constitute a major barrier to access to advanced biological therapies, especially in low- and middle-income countries. By reducing the cost of biosimilars through supportive policies and incentives, more patients can benefit from high-quality, affordable treatments and ultimately improve public health outcomes.
- International collaboration and support for developing countries: International support and collaboration in the development and use of biosimilars are essential to improve access to biological medicines, mainly in developing countries. This is achieved through technology transfer, training, strategic alliances, regulatory support, and financing of research projects. These actions not only help reduce costs and improve the availability of treatments for serious diseases such as cancer but also strengthen the capacity of these countries to produce and regulate high-quality biosimilars in a sustainable manner.
  – Recommendation: Strengthen international cooperation to support infrastructure and regulatory capacity in developing countries, allowing them to fully benefit from biosimilars.
    * Actions: Establish international training programs to develop regulatory expertise in developing countries. Create twinning agreements in which regulatory agencies in developed countries advise their counterparts in developing regions. Provide technical assistance and funding to improve regulatory infrastructure and laboratory facilities. Facilitate the exchange of knowledge and best practices through international forums and collaborative networks.
    * Justification: Developing countries often lack the resources and expertise to effectively regulate and monitor the introduction of biosimilars. International support can help these countries establish strong regulatory frameworks, ensuring that biosimilars are safe, effective, and accessible to those who need them. Improving regulatory capacity will also attract investment in local production of biosimilars, fostering economic growth and improving healthcare outcomes.
- Patient and physician opinion: Patient and physician opinion on the use of biosimilars in cancer treatment is generally positive once initial mistrust is overcome through education and clinical experience. The different patient associations do a remarkable job informing patients. On the other hand, patients and health insurance associations value the reduction in costs and greater access to treatments, while physicians appreciate the comparable efficacy and economic benefits of biosimilars. However, both patients and physicians highlight the importance of clear regulation and continued education to maximize the adoption and success of biosimilars in clinical practice.
  – Recommendation: Develop comprehensive educational programs for physicians and patients that clarify pharmacist substitution policies. Incorporate the different associations into the discussion.
    * Actions: Implement targeted educational initiatives focused on the safety, efficacy, and immunogenicity of biosimilars, supported by current clinical trial data and real-world evidence. Develop clear policies on the pharmaceutical substitution of biologicals with biosimilars, guaranteeing transparency and communication.
    * Justification: Better education will close knowledge gaps, build clinician confidence, and address concerns about the use of biosimilars, thereby promoting their broader prescription and integration into clinical practice. Clear communication can alleviate concerns and build trust, supporting the wider adoption of biosimilars.

The development and adoption of biosimilars is crucial to providing high-quality, affordable healthcare solutions worldwide. Implementing harmonized regulatory frameworks, using artificial intelligence technologies, and promoting clinical evidence are essential steps to realize the full potential of biosimilars to improve public health outcomes. It is vital to establish pricing and reimbursement policies that encourage the adoption of biosimilars, especially in developing countries. Strengthening international cooperation to support infrastructure and regulatory capacity in developing countries will allow them to fully benefit from biosimilars. This comprehensive approach will facilitate the global adoption of biosimilars, improve access to advanced treatments, and reduce healthcare costs. Develop comprehensive educational modules for healthcare providers, and patient and treating physician associations, and organize international conferences to share trial results and real-world data on the efficacy and safety of biosimilars. Better education and favorable policies can increase the confidence and adoption of these medications among healthcare professionals.

## 9. Challenges and Future Considerations

Despite its numerous benefits, the implementation of AI in biosimilar development presents challenges, including the need for large, high-quality data sets to train algorithms, concerns about data privacy, and the need for careful interpretation of the results generated by AI. Furthermore, the acceptance of AI-based techniques by regulatory authorities is still evolving, which requires continued dialogue between drug developers and regulators to ensure that AI innovations are effectively and safely integrated into regulatory standards. Artificial intelligence is shaping a new paradigm in biosimilar development, one that promises to significantly improve the efficacy, safety, and accessibility of these essential therapies. As technology continues to advance, we are likely to see even more innovative applications that will transform the biopharmaceutical industry.

## Figures and Tables

**Table 1:** Total number of biosimilars approved until 2023 by the eight countries analyzed.

Countries and Sources	Total Number Approved	Regulatory Framework for Biosimilars
Canada [134]	53 (September 2023)	CMA
Brazil [135]	52 (May 2023)	ANVISA
United States [136]	45 (December 2023)	FDA
Australia [137]	43 (September 2023)	TGA
Japan [138]	32 (December 2022)	PMDA
South Korea [108]	25 (December 2022)	MFDS
Argentina [126]	24 (December 2022)	ANMAT
South Africa [139]	5 (November 2020)	SAHPRA

## Appendix A

Table A1 of the role of NLP in medical studies: M-FLAG: medical vision–language pre-training with frozen language models and latent space geometry optimization; the frozen language model helps ECG zero-shot learning; Med-UniC: unifying cross-lingual medical vision–language pre-training by a diminishing bias.

**Table A1:** The role of NLP in medical studies.

Study	Main Contribution	Methodology	Significance
M-FLAG: medical vision–language pre-training with frozen language models and latent space geometry optimization [205].	Introduces M-FLAG, a model that combines frozen language models with vision–language pre-training for medical applications.	Utilizes frozen language models to maintain robust linguistic features. Optimizes latent space geometry to enhance alignment between visual and textual information.	Improves medical image understanding and diagnostic capabilities. Facilitates more accurate and interpretable medical AI applications.
Frozen language model helps ECG zero-shot learning [206].	Demonstrates the effectiveness of frozen language models in performing zero-shot learning on ECG data.	Applies a pre-trained language model to interpret ECG signals without additional training on ECG- specific data. Leverages the generalization ability of language models to understand medical terminology and concepts.	Enables rapid deployment of ECG analysis tools without the need for extensive domain-specific data. Enhances the ability of AI to generalize across different medical datasets and tasks.
Med-UniC: unifying cross-lingual medical vision–language pre-training by diminishing bias [207].	Proposes Med-UniC, a model that addresses cross-lingual and cross-modal biases in medical vision–language pre-training.	Implements techniques to diminish biases present in multilingual and multimodal medical datasets. Uses a unified framework to align vision and language representations across different languages and medical contexts.	Promotes more equitable AI tools that perform consistently across diverse patient populations. Enhances the usability of medical AI in multilingual and multicultural healthcare settings.

## References

[1] Aggarwal G., Nagpal M., Sharma A., Puri V., Dhingra G.A. (2021). Upcoming Drifts in Bio-similars. Curr. Rev. Clin. Exp. Pharmacol..

[2] Conlé M. (2019). Recent developments in China’s biopharmaceutical industry (2012–2017): Patterns of product innovation and firm scope. J. Sci. Technol. Policy Manag..

[3] González-Ramírez R., Castañeda-Hernández G. (2019). The challenges of developing and commercializing biosimilars in Latin America. Pharm. Pat. Anal..

[4] Rose S.A., Rice T. The Biosimilar Action Plan: An Effective Mechanism for Balancing Biologic Innovation and Competition in the United States? (18 November 2019). McGeorge Law Review, Forthcoming, Wake Forest Univ. Legal Studies Paper. https://ssrn.com/abstract=3489444.

[5] Akram M.S., Pery N., Butler L., Shafiq M.I., Batool N., Rehman M.F.U., Grahame-Dunn L.G., Yetisen A.K. (2021). Challenges for biosimilars: Focus on rheumatoid arthritis. Crit. Rev. Biotechnol..

[6] Eniu A., Cherny N.I., Bertram M., Thongprasert S., Douillard J.Y., Bricalli G., Vyas M., Trapani D. (2019). Cancer medicines in Asia and Asia-Pacific: What is available, and is it effective enough?. ESMO Open.

[7] Santos S.B., Sousa Lobo J.M., Silva A.C. (2019). Biosimilar medicines used for cancer therapy in Europe: A review. Drug Discov. Today.

[8] Annett S. (2021). Pharmaceutical drug development: High drug prices and the hidden role of public funding. Biol. Futur..

[9] Bourgeron T., Geiger S. (2022). (De-)assetizing pharmaceutical patents: Patent contestations behind a blockbuster drug. Econ. Soc..

[10] Nicholson Price W. The Cost of Novelty (March 11, 2019). 120 Colum. L. Rev. 769 (2020), U of Michigan Public Law Research Paper No. 633, U of Michigan Law & Econ Research Paper No. 19-004. https://ssrn.com/abstract=3350477.

[11] Kabir E.R., Moreino S.S., Sharif Siam M.K. (2019). The Breakthrough of Biosimilars: A Twist in the Narrative of Biological Therapy. Biomolecules.

[12] Bas T.G. (2023). Biosimilars for the next decade in Latin America: A window of opportunity. Expert Opin. Biol. Ther..

[13] Li P., Zheng Y., Chen X. (2017). Drugs for Autoimmune Inflammatory Diseases: From Small Molecule Compounds to Anti-TNF Biologics. Front. Pharmacol..

[14] Koźmiński P., Halik P.K., Chesori R., Gniazdowska E. (2020). Overview of Dual-Acting Drug Methotrexate in Different Neurological Diseases, Autoimmune Pathologies and Cancers. Int. J. Mol. Sci..

[15] Najeeb H., Yasmin F., Surani S. (2022). Emerging role of biosimilars in the clinical care of inflammatory bowel disease patients. World J. Clin. Cases.

[16] Leufkens H.G., Kusynová Z., Aitken M., Hoekman J., Stolk P., Klein K., Mantel-Teeuwisse A.K. (2022). Four scenarios for the future of medicines and social policy in 2030. Drug Discov. Today.

[17] Wasan H., Singh D., Reeta K.H., Gupta P., Gupta Y.K. (2022). Drug development process and COVID-19 pandemic: Flourishing era of outsourcing. Indian J. Pharmacol..

[18] Oriama R., Mudida R., Burger-Helmchen T., Rezaei N. (2022). A Multi-level Perspective to Biosimilars Development: Pathways Towards Incremental Innovation in the Health Bioeconomy. Transdisciplinarity.

[19] Agbogbo F.K., Ecker D.M., Farrand A., Han K., Khoury A., Martin A., McCool J., Rasche U., Rau T.D., Schmidt D. (2019). Current perspectives on biosimilars. J. Ind. Microbiol. Biotechnol..

[20] Sayah R., Awada S., Ismaiil L., Hatem G. (2023). Assessment of the differences between generic and biosimilar drugs: A brief literature review. J. Generic Med..

[21] Abraham I. (2023). Preparing for the third decade of biosimilars. Expert Opin. Biol. Ther..

[22] Hobbs A.L., Crawford J.P. (2019). Biosimilars and implications for pharmacy practice: Ready or not, here they come!. Pharm. Pract..

[23] Ratih R., Asmari M., Abdel-Megied A.M., Elbarbry F., El Deeb S. (2021). Biosimilars: Review of regulatory, manufacturing, analytical aspects and beyond. Microchem. J..

[24] Kang H.N., Thorpe R., Knezevic I., Casas Levano M., Chilufya M.B., Chirachanakul P., Chua H.M., Dalili D., Foo F., Gao K. (2021). Regulatory challenges with biosimilars: An update from 20 countries. Ann. N. Y. Acad. Sci..

[25] Rathore A.S., Bhargava A. (2021). Regulatory considerations in biosimilars: Latin America region. Prep. Biochem. Biotechnol..

[26] O’Callaghan J., Barry S.P., Bermingham M., Morris J.M., Griffin B.T. (2019). Regulation of biosimilar medicines and current perspectives on interchangeability and policy. Eur. J. Clin. Pharmacol..

[27] Rahalkar H., Sheppard A., Salek S. (2022). Biosimilar development and review process in the BRICS-TM countries: Proposal for a standardized model to improve regulatory performance. Expert Rev. Clin. Pharmacol..

[28] Dhiman S.K., Dureja H. (2021). Comparative analysis of evolution of regulatory environment in USA, Europe and Japan. Pharma Innov..

[29] Chhabra H., Mouslim M.C., Kashiramka S., Rathore A.S. (2022). Dynamics of biosimilar uptake in emerging markets. Expert Opin. Biol. Ther..

[30] Sadek M.A.Z. (2020). Global Status of Biosimilars and Its Influential Factors. Eur. J. Bus. Manag. Res..

[31] Wadhwa M., Kang H.N., Jivapaisarnpong T., Andalucia L.R., Blades C.D.R.Z., Casas Levano M., Chang W., Chew J.Y., Chilufya M.B., Chirachanakul P. (2020). WHO implementation workshop on guidelines on procedures and data requirements for changes to approved biotherapeutic products, Seoul, Republic of Korea, 25–26 June 2019. Biologicals.

[32] Ortiz-Prado E., Ponce-Zea J., Vasconez J.E., Castillo D., Checa-Jaramilloz D.C., Rodríguez-Burneo N., Andrade F., Intriago-Baldeón D.P., Galarza-Maldonado C. (2020). Current trends for biosimilars in the Latin American market. Generics Biosimilars Initiat. J..

[33] Araujo R.L., Mosegui G.B.G., Vianna C.M.D.E.M., Villar F.A., Catao T.P. (2020). Reflections and Perspectives on Biosimilars in Brazil. Int. J. Pharm. Pharm. Sci..

[34] Moeti L., Litedu M., Joubert J. (2023). The Implementation of a Risk-Based Assessment Approach by the South African Health Products Regulatory Authority (SAHPRA). Pharm. Med..

[35] Kolluri S., Lin J., Liu R., Zhang Y., Zhang W. (2022). Machine Learning and Artificial Intelligence in Pharmaceutical Research and Development: A Review. AAPS J..

[36] Puranik A., Dandekar P., Jain R. (2022). Exploring the potential of machine learning for more efficient development and production of biopharmaceuticals. Biotechnol. Prog..

[37] Niazi S.K. (2022). Molecular Biosimilarity—An AI-Driven Paradigm Shift. Int. J. Mol. Sci..

[38] Lamanna W.C., Holzmann J., Cohen H.P., Guo X., Schweigler M., Stangler T., Seidl A., Schiestl M. (2018). Maintaining consistent quality and clinical performance of biopharmaceuticals. Expert Opin. Biol. Ther..

[39] Huggins D.J., Biggin P.C., Dämgen M.A., Essex J.W., Harris S.A., Henchman R.H., Khalid S., Kuzmanic A., Laughton C.A., Michel J. (2019). Biomolecular simulations: From dynamics and mechanisms to computational assays of biological activity. WIREs Comput. Mol. Sci..

[40] Vamathevan J., Clark D., Czodrowski P., Dunham I., Ferran E., Lee G., Li B., Madabhushi A., Shah P., Spitzer M. (2019). Applications of machine learning in drug discovery and development. Nat. Rev. Drug Discov..

[41] Wang J., Deng H., Liu B., Hu A., Liang J., Fan L., Zheng X., Wang T., Lei J. (2020). Systematic Evaluation of Research Progress on Natural Language Processing in Medicine Over the Past 20 Years: Bibliometric Study on PubMed. J. Med. Internet Res..

[42] Galo E.B., Patricio G.B., Andres D.P., Silvana L.G., María C.M.-C., Inamuddin, Altalhi T., Cruz J.N., Refat M.S.E. (2022). Machine Learning Approaches to Improve Prediction of Target-Drug Interactions. Drug Design Using Machine Learning.

[43] Malakar S., Gontor E.N., Dugbaye M.Y., Shah K., Sinha S., Sutaoney P., Chauhan N.S. (2024). Cancer treatment with biosimilar drugs: A review. Cancer Innov..

[44] Bachu R.D., Abou-Dahech M., Balaji S., Boddu S.H.S., Amos S., Singh V., Babu R.J., Tiwari A.K. (2022). Oncology biosimilars: New developments and future directions. Cancer Rep..

[45] Ditani A.S., Mallick P.P., Anup N., Tambe V., Polaka S., Sengupta P., Rajpoot K., Tekade R.K. (2021). Biosimilars accessible in the market for the treatment of cancer. J. Control. Release.

[46] Peña A.N., Rodríguez C.D.C. (2021). Requirements for Biosimilar Authorization: A Legal and Comparative Perspective—FDA Versus EMA. Curr. Sci..

[47] Bas T.G., Sáez M.L., Sáez N. (2024). Sustainable Development versus Extractivist Deforestation in Tropical, Subtropical, and Boreal Forest Ecosystems: Repercussions and Controversies about the Mother Tree and the Mycorrhizal Network Hypothesis. Plants.

[48] Barros M.V., Salvador R., Do Prado G.F., De Francisco A.C., Piekarski C.M. (2021). Circular economy as a driver to sustainable businesses. Clean. Environ. Syst..

[49] Gusenbauer M., Haddaway N.R. (2020). Which academic search systems are suitable for systematic reviews or meta-analyses? Evaluating retrieval qualities of Google Scholar, PubMed, and 26 other resources. Res. Synth. Methods.

[50] Scells H., Zuccon G., Koopman B., Clark J. Automatic Boolean Query Formulation for Systematic Review Literature Search. Proceedings of the Web Conference 2020.

[51] Scells H., Zuccon G., Koopman B. (2021). A comparison of automatic Boolean query formulation for systematic reviews. Inf. Retr. J..

[52] Lashkari F., Ensan F., Bagheri E., Ghorbani A.A. (2017). Efficient indexing for semantic search. Expert Syst. Appl..

[53] Rugo H.S., Rifkin R.M., Declerck P., Bair A.H., Morgan G. (2019). Demystifying biosimilars: Development, regulation and clinical use. Future Oncol..

[54] Joshi S., Rathore A.S. (2020). Assessment of Structural and Functional Comparability of Biosimilar Products: Trastuzumab as a Case Study. Biodrugs Clin. Immunother. Biopharm. Gene Ther..

[55] Genovese M.C., Sanchez-Burson J., Oh M., Balazs E., Neal J., Everding A., Hala T., Wojciechowski R., Fanjiang G., Cohen S. (2020). Comparative clinical efficacy and safety of the proposed biosimilar ABP 710 with infliximab reference product in patients with rheumatoid arthritis. Arthritis Res. Ther..

[56] Rathore A.S., Stevenson J.G., Chhabra H. (2021). Considerations related to comparative clinical studies for biosimilars. Expert Opin. Drug Saf..

[57] Bielsky M.C., Cook A., Wallington A., Exley A., Kauser S., Hay J.L., Both L., Brown D. (2020). Streamlined approval of biosimilars: Moving on from the confirmatory efficacy trial. Drug Discov. Today.

[58] Moore T.J., Mouslim M.C., Blunt J.L., Alexander G.C., Shermock K.M. (2021). Assessment of Availability, Clinical Testing, and US Food and Drug Administration Review of Biosimilar Biologic Products. JAMA Intern. Med..

[59] Kumar R., Guttman A., Rathore A.S. (2022). Applications of capillary electrophoresis for biopharmaceutical product characterization. Electrophoresis.

[60] Reardon G. (2020). Pharmacoeconomics of Biologic Medicines and Biosimilars. Biologics, Biosimilars, and Biobetters.

[61] Rezk M.F., Pieper B. (2018). To See or NOsee: The Debate on the Nocebo Effect and Optimizing the Use of Biosimilars. Adv. Ther..

[62] Colloca L., Panaccione R., Murphy T.K. (2019). The Clinical Implications of Nocebo Effects for Biosimilar Therapy. Front. Pharmacol..

[63] Grosso F., Barbiani D., Cavalera C., Volpato E., Pagnini F. (2024). Risk factors associated with nocebo effects: A review of reviews. Brain Behav. Immun. Health.

[64] Colloca L. (2024). The Nocebo Effect. Annu. Rev. Pharmacol. Toxicol..

[65] Wu Q., Wang Z., Wang X., Yu H., Sun J. (2023). Patients’ Perceptions of Biosimilars: A Systematic Review. BioDrugs.

[66] Gibofsky A., Jacobson G., Franklin A., O’Hara-Levi S., Peyrin-Biroulet L., McGrath M., McCabe D. (2023). An online survey among US patients with immune-mediated conditions: Attitudes about biosimilars. J. Manag. Care Spec. Pharm..

[67] Wojtukiewicz M.Z., Politynska B., Skalij P., Tokajuk P., Wojtukiewicz A.M., Honn K.V. (2019). It is not just the drugs that matter: The nocebo effect. Cancer Metastasis Rev..

[68] McGarvey N., Gitlin M., Fadli E., Chung K.C. (2022). Increased healthcare costs by later stage cancer diagnosis. BMC Health Serv. Res..

[69] Seidman J., Masi D., Gomez-Rexrode A.E. (2019). Personalizing Value in Cancer Care: The Case for Incorporating Patient Preferences Into Routine Clinical Decision Making. J. Particip. Med..

[70] Oehrlein E.M., Harris J., Balch A., Furlong P., Hargis E., Woolley M., Perfetto E. (2021). Improving Access and Quality of Health Care in the United States: Shared Goals Among Patient Advocates. Patient Patient-Centered Outcomes Res..

[71] Feldman D., Kruger P., Delbecque L., Duenas A., Bernard-Poenaru O., Wollenschneider S., Hicks N., Reed J.A., Sargeant I., Pakarinen C. (2021). Co-creation of practical “how-to guides” for patient engagement in key phases of medicines development—From theory to implementation. Res. Involv. Engagem..

[72] Coylewright M., Otero D., Lindman B.R., Levack M.M., Horne A., Ngo L.H., Beaudry M., Col H.V., Col N.F. (2024). An interactive, online decision aid assessing patient goals and preferences for treatment of aortic stenosis to support physician-led shared decision-making: Early feasibility pilot study. PLoS ONE.

[73] Rosembert D.C., Twigg M.J., Wright D.J. (2024). Patient’s and Consultant’s Views and Perceptions on Switching from an Originator Biologic to Biosimilar Medication: A Qualitative Study. Pharmacy.

[74] Varma M., Almarsdóttir A.B., Druedahl L.C. (2022). “Biosimilar, so it looks alike, but what does it mean?” A qualitative study of Danish patients’ perceptions of biosimilars. Basic Clin. Pharmacol. Toxicol..

[75] Sarnola K., Merikoski M., Jyrkkä J., Hämeen-Anttila K. (2020). Physicians’ perceptions of the uptake of biosimilars: A systematic review. BMJ Open.

[76] Druedahl L.C., Kälvemark Sporrong S., Van De Weert M., De Bruin M.L., Hoogland H., Minssen T., Almarsdóttir A.B. (2021). Evolving Biosimilar Clinical Requirements: A Qualitative Interview Study with Industry Experts and European National Medicines Agency Regulators. BioDrugs.

[77] Barbier L., Mbuaki A., Simoens S., Declerck P., Vulto A.G., Huys I. (2022). Regulatory Information and Guidance on Biosimilars and Their Use Across Europe: A Call for Strengthened One Voice Messaging. Front. Med..

[78] Foreman E., Patel H., Siderov J., Harchowal J., Bubalo J., Chan A. (2020). A survey of global biosimilar implementation practice conducted by the International Society of Oncology Pharmacy Practitioners. J. Oncol. Pharm. Pract..

[79] Mulcahy A.W., Hlavka J.P., Case S.R. (2018). Biosimilar Cost Savings in the United States: Initial Experience and Future Potential. Rand Health Q..

[80] Yang J., Liu R., Ektare V., Stephens J., Shelbaya A. (2021). Does Biosimilar Bevacizumab Offer Affordable Treatment Options for Cancer Patients in the USA? A Budget Impact Analysis from US Commercial and Medicare Payer Perspectives. Appl. Health Econ. Health Policy.

[81] Van De Wiele V.L., Kesselheim A.S., Sarpatwari A. (2021). Barriers To US Biosimilar Market Growth: Lessons From Biosimilar Patent Litigation: Article examines barriers to US biosimilar market growth. Health Aff..

[82] Nabhan C., Parsad S., Mato A.R., Feinberg B.A. (2018). Biosimilars in Oncology in the United States: A Review. JAMA Oncol..

[83] Baumgart D.C., Misery L., Naeyaert S., Taylor P.C. (2019). Biological Therapies in Immune-Mediated Inflammatory Diseases: Can Biosimilars Reduce Access Inequities?. Front. Pharmacol..

[84] Dutta B., Huys I., Vulto A.G., Simoens S. (2020). Identifying Key Benefits in European Off-Patent Biologics and Biosimilar Markets: It is Not Only About Price!. BioDrugs.

[85] Alnaqbi K.A., Bellanger A., Brill A., Castañeda-Hernández G., Clopés Estela A., Delgado Sánchez O., García-Alfonso P., Gyger P., Heinrich D., Hezard G. (2023). An international comparative analysis and roadmap to sustainable biosimilar markets. Front. Pharmacol..

[86] Moorkens E., Vulto A.G., Huys I. (2021). Biosimilars in Belgium: A proposal for a more competitive market. Acta Clin. Belg..

[87] Simoens S., Lacosta T.B., Inotai A. (2024). Learnings from cross-border biosimilar pricing policies in Europe. Expert Rev. Pharmacoecon. Outcomes Res..

[88] Tachkov K., Savova A., Manova M., Petrova G. (2023). Tackling reimbursement challenges to fair access to medicines – introduction to the topic. Expert Rev. Pharmacoecon. Outcomes Res..

[89] Simoens S., Cheung R. (2020). Tendering and biosimilars: What role for value-added services?. J. Mark. Access Health Policy.

[90] Oskouei S.T., Kusmierczyk A.R. (2021). Biosimilar Uptake: The Importance of Healthcare Provider Education. Pharm. Med..

[91] Leonard E., Wascovich M., Oskouei S., Gurz P., Carpenter D. (2019). Factors Affecting Health Care Provider Knowledge and Acceptance of Biosimilar Medicines: A Systematic Review. J. Manag. Care Spec. Pharm..

[92] Dalpoas S.E., Socal M., Proctor C., Shermock K.M. (2020). Barriers to biosimilar utilization in the United States. Am. J. Health-Syst. Pharm..

[93] Panda S., Singh P.K., Mishra S., Mitra S., Pattnaik P., Adhikary S.D., Mohapatra R.K. (2023). Indian Biosimilars and Vaccines at Crossroads–Replicating the Success of Pharmagenerics. Vaccines.

[94] Perpoil A., Grimandi G., Birklé S., Simonet J.F., Chiffoleau A., Bocquet F. (2020). Public Health Impact of Using Biosimilars, Is Automated Follow up Relevant?. Int. J. Environ. Res. Public Health.

[95] Vandenplas Y., Simoens S., Van Wilder P., Vulto A.G., Huys I. (2021). Informing Patients about Biosimilar Medicines: The Role of European Patient Associations. Pharmaceuticals.

[96] Blanken M.O., Frederix G.W., Nibbelke E.E., Koffijberg H., Sanders E.A.M., Rovers M.M., Bont L. (2018). Cost-effectiveness of rule-based immunoprophylaxis against respiratory syncytial virus infections in preterm infants. Eur. J. Pediatr..

[97] Kim H., Alten R., Avedano L., Dignass A., Gomollón F., Greveson K., Halfvarson J., Irving P.M., Jahnsen J., Lakatos P.L. (2020). The Future of Biosimilars: Maximizing Benefits Across Immune-Mediated Inflammatory Diseases. Drugs.

[98] Goeree R., Chiva-Razavi S., Gunda P., Jain M., Jugl S. (2019). Cost-effectiveness analysis of secukinumab in ankylosing spondylitis from the Canadian perspective. J. Med. Econ..

[99] Olfatifar M., Aghdaei H.A., Dehkordi A.H., Shahrokh S., Pourhoseingholi M.A., Balaii H., Rajabnia M., Ivanchuk M., Ivanchuk P., Nazari S.H. (2022). Cost-effectiveness analysis of infliximab versus CinnoRA in the treatment of moderate to severe ulcerative colitis in Iranian patients. Immunopathol. Persa.

[100] Dilokthornsakul P., Sawangjit R., Osiri M., Chiowchanwisawakit P., Louthrenoo W., Permsuwan U. (2023). Cost-Utility Analysis of Biologic Disease-Modifying Antirheumatic Drugs for Patients With Psoriatic Arthritis in Thailand. Value Health Reg. Issues.

[101] Vogler S., Schneider P., Zuba M., Busse R., Panteli D. (2021). Policies to Encourage the Use of Biosimilars in European Countries and Their Potential Impact on Pharmaceutical Expenditure. Front. Pharmacol..

[102] Humphreys S.Z. (2022). Real-World Evidence of a Successful Biosimilar Adoption Program. Future Oncol..

[103] Mestre-Ferrandiz J., Czech M., Smolen J.S., Cornes P., Aapro M.S., Danese S., Deitch S., Tyldsley H., Foster W., Shah P. (2024). Capturing the holistic value of biosimilars in Europe—Part 1: A historical perspective. Expert Rev. Pharmacoecon. Outcomes Res..

[104] Yousefi N., Ahmadi R., Tayeba H., Taheri S., Mahboudi F., Peiravian F. (2020). Biosimilar Medicines in the Iranian Market: A Way to More Affordable Medicines. Indian J. Pharm. Sci..

[105] Brian G., Biljana T., Eleonora A., Magdalene W., Stuart M., Amanj K., Mainul H., Sean M.S., Francis K., Amos M. (2022). Biosimilars are essential for sustainable healthcare systems; however, key challenges remain as seen with long-acting insulin analogues. J. Appl. Pharm. Sci..

[106] Bas T.G., Oliu C.A. (2018). Innovation strategy management survey of the Chilean biomedical industry. Assessment of windows of opportunities to reduce technological gaps. Int. J. Health Plan. Manag..

[107] Esteban E., Bustos R.H., García J.C., Jáuregui E. (2019). Biosimilars: An Approach to some Current Worldwide Regulation Frameworks. Curr. Clin. Pharmacol..

[108] Klein K., Gencoglu M., Heisterberg J., Acha V., Stolk P. (2023). The Global Landscape of Manufacturers of Follow-on Biologics: An Overview of Five Major Biosimilar Markets and 15 Countries. BioDrugs.

[109] Sheth S., Vashishta L., Goyal R., Rathore A., Chirmule N. (2023). On the Manufacturers of Biosimilars in Asia. Clin. Pharmacol. Ther..

[110] Darrow J.J., Avorn J., Kesselheim A.S. (2020). FDA Approval and Regulation of Pharmaceuticals, 1983–2018. JAMA.

[111] Goode R., Chao B. (2022). Biological patent thickets and delayed access to biosimilars, an American problem. J. Law Biosci..

[112] Kang H.N., Thorpe R., Knezevic I., Survey Participants from 19 Countries (2020). The regulatory landscape of biosimilars: WHO efforts and progress made from 2009 to 2019. Biol. J. Int. Assoc. Biol. Stand..

[113] Yazdany J. (2020). Failure to Launch: Biosimilar Sales Continue to Fall Flat in the United States. Arthritis Rheumatol..

[114] Halimi V., Daci A., Ancevska Netkovska K., Suturkova L., Babar Z.U.D., Grozdanova A. (2020). Clinical and Regulatory Concerns of Biosimilars: A Review of Literature. Int. J. Environ. Res. Public Health.

[115] Brown D.G., Wobst H.J. (2021). A Decade of FDA-Approved Drugs (2010–2019): Trends and Future Directions. J. Med. Chem..

[116] Carrier M.A., Tu S. Why Pharmaceutical Patent Thickets Are Unique (August 1, 2023). Texas Intellectual Property Law Journal, Forthcoming, Rutgers Law School Research Paper, WVU College of Law Research Paper No. 2023-25. https://ssrn.com/abstract=4571486.

[117] Reilly M.S., Schneider P.J. (2021). A critical review of substitution policy for biosimilars in Canada. Generics Biosimilars Initiat. J..

[118] Rathore A.S., Bhargava A. (2020). Biosimilars in Developed Economies: Overview, Status, and Regulatory Considerations. Regul. Toxicol. Pharmacol..

[119] Murdoch B., Caulfield T. (2020). The Law and Ethics of Switching from Biologic to Biosimilar in Canada. J. Can. Assoc. Gastroenterol..

[120] Bennett C.L., Schoen M.W., Hoque S., Witherspoon B.J., Aboulafia D.M., Hwang C.S., Ray P., Yarnold P.R., Chen B.K., Schooley B. (2020). Improving oncology biosimilar launches in the EU, the USA, and Japan: An updated Policy Review from the Southern Network on Adverse Reactions. Lancet. Oncol..

[121] Punia A., Malhotra H., Ramzan I. (2020). International Regulatory Processes and Policies for Innovator Biologics, Biosimilars, and Biobetters. Biologics, Biosimilars, and Biobetters.

[122] Na S.Y., Choi C.H., Song E.M., Bang K.B., Park S.H., Kim E.S., Park J.J., Keum B., Lee C.K., Lee B.I. (2023). Korean clinical practice guidelines on biologics and small molecules for moderate-to-severe ulcerative colitis. Intest. Res..

[123] Son K.B. (2021). Market Exclusivity of the Originator Drugs in South Korea: A Retrospective Cohort Study. Front. Public Health.

[124] Babu D.N.A. (2023). Biosimilars The Emerging Economical Oncotherapy.

[125] Gregory G.P., Carrington C., Cheah C.Y., Hawkes E.A., Irving I.M., Siderov J., Opat S. (2020). A consensus statement on the use of biosimilar medicines in hematology in Australia. Asia-Pac. J. Clin. Oncol..

[126] Da Silva Machado F.L., Cañás M., Doubova S.V., Urtasun M.A., Marín G.H., Osorio-de Castro C.G.S., Albuquerque F.C., Ribeiro T.B., Pont L., Crisóstomo Landeros J. (2023). Biosimilars approvals by thirteen regulatory authorities: A cross-national comparison. Regul. Toxicol. Pharmacol..

[127] Deeksha K.S., Veeranna B., Ravindra G.K. (2023). Analyzing Biosimilars in Brazil: Comprehensive Specifications of the Regulatory System. Indian J. Pharm. Educ. Res..

[128] Prasanthi Nori L., Ravi P. (2020). Comparability Pathway for the Approval of Similar Biologics with Respect to Reference Biologics in Europe and Brazil. Indian J. Pharm. Educ. Res..

[129] Abu-Zaid M.H., Adebajo A., Miedany Y.E. (2023). Potential for biosimilars in rheumatology in Africa. Ann. Rheum. Dis..

[130] Pategou J. (2020). Africa’s Biosimilar Landscape: Outlook & Current Challenges. https://www.biosimilardevelopment.com/doc/africa-s-biosimilar-landscape-outlook-current-challenges-0001.

[131] Kale D. (2019). From small molecule generics to biosimilars: Technological upgrading and patterns of distinctive learning processes in the Indian pharmaceutical industry. Technol. Forecast. Soc. Chang..

[132] Dureja H., Dhiman S. (2021). SAHPRA—Relevance of the New South African Health Products Regulatory Authority and Opportunities Ahead. J. Regul. Sci..

[133] Ncube B.M., Dube A., Ward K. (2021). Establishment of the African Medicines Agency: Progress, challenges and regulatory readiness. J. Pharm. Policy Pract..

[134] Smart & Biggar (2022). Biosimilars Approved in Canada. https://www.smartbiggar.ca/insights/biosimilars.

[135] Cestari de Oliveira S.H. (2023). Follow-on biologicals/biosimilars approved in Brazil: May 2023 update—GaBI Journal. Generics Biosimilars Initiat. J..

[136] Stewart J. (2024). What Biosimilars Have Been Approved in the United States?. https://www.drugs.com/medical-answers/many-biosimilars-approved-united-states-3463281/.

[137] Jeremias S. (2023). Regulatory Updates from around the Globe Provide Hope for Biosimilars. https://www.centerforbiosimilars.com/view/regulatory-updates-from-around-the-globe-provide-hope-for-biosimilars.

[138] Kuribayashi R., Nakano A., Hariu A., Kishioka Y., Honda F. (2023). Historical Overview of Regulatory Approvals and PMDA Assessments for Biosimilar Products in Japan During 2009–2022. BioDrugs.

[139] Jeremias S. (2020). Alvotech and Cipla Partner on 5 Biosimilars for South Africa. https://www.centerforbiosimilars.com/view/alvotech-and-cipla-partner-on-5-biosimilars-for-south-africa.

[140] Opderbeck D. (2019). Artificial Intelligence in Pharmaceuticals, Biologics, and Medical Devices: Present and Future Regulatory Models. Fordham Law Rev..

[141] Zohuri B., Behgounia F. (2023). Application of artificial intelligence driving nano-based drug delivery system. A Handbook of Artificial Intelligence in Drug Delivery.

[142] Askari S., Ghofrani A., Taherdoost H. (2023). Transforming Drug Design: Innovations in Computer-Aided Discovery for Biosimilar Agents. BioMedInformatics.

[143] Pandya S., Thakur A., Saxena S., Jassal N., Patel C., Modi K., Shah P., Joshi R., Gonge S., Kadam K. (2021). A Study of the Recent Trends of Immunology: Key Challenges, Domains, Applications, Datasets, and Future Directions. Sensors.

[144] Walsh I., Myint M., Nguyen-Khuong T., Ho Y.S., Ng S.K., Lakshmanan M. (2022). Harnessing the potential of machine learning for advancing “Quality by Design” in biomanufacturing. mAbs.

[145] Sharma D.K., Bhargava S., Singhal K. (2020). Internet of Things applications in the pharmaceutical industry. An Industrial IoT Approach for Pharmaceutical Industry Growth.

[146] Agamah F.E., Mazandu G.K., Hassan R., Bope C.D., Thomford N.E., Ghansah A., Chimusa E.R. (2020). Computational/in silico methods in drug target and lead prediction. Briefings Bioinform..

[147] Viceconti M., Pappalardo F., Rodriguez B., Horner M., Bischoff J., Musuamba Tshinanu F. (2021). In silico trials: Verification, validation and uncertainty quantification of predictive models used in the regulatory evaluation of biomedical products. Methods.

[148] Rathore A.S., Nikita S., Thakur G., Mishra S. (2023). Artificial intelligence and machine learning applications in biopharmaceutical manufacturing. Trends Biotechnol..

[149] Nupur N., Joshi S., Gulliarme D., Rathore A.S. (2022). Analytical Similarity Assessment of Biosimilars: Global Regulatory Landscape, Recent Studies and Major Advancements in Orthogonal Platforms. Front. Bioeng. Biotechnol..

[150] Alarcón-Zendejas A.P., Scavuzzo A., Jiménez-Ríos M.A., Álvarez Gómez R.M., Montiel-Manríquez R., Castro-Hernández C., Jiménez-Dávila M.A., Pérez-Montiel D., González-Barrios R., Jiménez-Trejo F. (2022). The promising role of new molecular biomarkers in prostate cancer: From coding and non-coding genes to artificial intelligence approaches. Prostate Cancer Prostatic Dis..

[151] Ghaffari Laleh N., Ligero M., Perez-Lopez R., Kather J.N. (2023). Facts and Hopes on the Use of Artificial Intelligence for Predictive Immunotherapy Biomarkers in Cancer. Clin. Cancer Res..

[152] Volovat S.R., Augustin I., Zob D., Boboc D., Amurariti F., Volovat C., Stefanescu C., Stolniceanu C.R., Ciocoiu M., Dumitras E.A. (2022). Use of Personalized Biomarkers in Metastatic Colorectal Cancer and the Impact of AI. Cancers.

[153] Kyriazakos S., Pnevmatikakis A., Cesario A., Kostopoulou K., Boldrini L., Valentini V., Scambia G. (2021). Discovering Composite Lifestyle Biomarkers With Artificial Intelligence From Clinical Studies to Enable Smart eHealth and Digital Therapeutic Services. Front. Digit. Health.

[154] Xiao Q., Zhang F., Xu L., Yue L., Kon O.L., Zhu Y., Guo T. (2021). High-throughput proteomics and AI for cancer biomarker discovery. Adv. Drug Deliv. Rev..

[155] Olivera P., Danese S., Jay N., Natoli G., Peyrin-Biroulet L. (2019). Big data in IBD: A look into the future. Nat. Rev. Gastroenterol. Hepatol..

[156] Castro Corredor D., Calvo Pascual L.A. (2023). Imbalanced machine learning classification models for removal biosimilar drugs and increased activity in patients with rheumatic diseases. PLoS ONE.

[157] Sarkar C., Das B., Rawat V.S., Wahlang J.B., Nongpiur A., Tiewsoh I., Lyngdoh N.M., Das D., Bidarolli M., Sony H.T. (2023). Artificial Intelligence and Machine Learning Technology Driven Modern Drug Discovery and Development. Int. J. Mol. Sci..

[158] Singh A.V., Ansari M.H.D., Rosenkranz D., Maharjan R.S., Kriegel F.L., Gandhi K., Kanase A., Singh R., Laux P., Luch A. (2020). Artificial Intelligence and Machine Learning in Computational Nanotoxicology: Unlocking and Empowering Nanomedicine. Adv. Healthc. Mater..

[159] Gupta R., Srivastava D., Sahu M., Tiwari S., Ambasta R.K., Kumar P. (2021). Artificial intelligence to deep learning: Machine intelligence approach for drug discovery. Mol. Divers..

[160] Krenn M., Ai Q., Barthel S., Carson N., Frei A., Frey N.C., Friederich P., Gaudin T., Gayle A.A., Jablonka K.M. (2022). SELFIES and the future of molecular string representations. Patterns.

[161] Brogi S., Ramalho T.C., Kuca K., Medina-Franco J.L., Valko M. (2020). Editorial: In silico Methods for Drug Design and Discovery. Front. Chem..

[162] Jiménez-Luna J., Grisoni F., Weskamp N., Schneider G. (2021). Artificial intelligence in drug discovery: Recent advances and future perspectives. Expert Opin. Drug Discov..

[163] Greener J.G., Kandathil S.M., Moffat L., Jones D.T. (2022). A guide to machine learning for biologists. Nat. Rev. Mol. Cell Biol..

[164] Tan P., Chen X., Zhang H., Wei Q., Luo K. (2023). Artificial intelligence aids in development of nanomedicines for cancer management. Semin. Cancer Biol..

[165] Noé F., Tkatchenko A., Müller K.R., Clementi C. (2020). Machine Learning for Molecular Simulation. Annu. Rev. Phys. Chem..

[166] Liu Y., Zhang D., Ren B., Gong X., Xu L., Feng Z.Q., Chang Y., He Y., Zheng J. (2020). Molecular simulations and understanding of antifouling zwitterionic polymer brushes. J. Mater. Chem. B.

[167] Temml V., Kutil Z. (2021). Structure-based molecular modeling in SAR analysis and lead optimization. Comput. Struct. Biotechnol. J..

[168] Lynch C.I., Rao S., Sansom M.S.P. (2020). Water in Nanopores and Biological Channels: A Molecular Simulation Perspective. Chem. Rev..

[169] Kurki P., Kang H.N., Ekman N., Knezevic I., Weise M., Wolff-Holz E. (2022). Regulatory Evaluation of Biosimilars: Refinement of Principles Based on the Scientific Evidence and Clinical Experience. BioDrugs.

[170] Woollett G.R., Park J.P., Han J., Jung B. (2023). The Role of PD Biomarkers in Biosimilar Development—To Get the Right Answer One Must First Ask the Right Question. Clin. Pharmacol. Ther..

[171] Terranova N., Renard D., Shahin M.H., Menon S., Cao Y., Hop C.E., Hayes S., Madrasi K., Stodtmann S., Tensfeldt T. (2024). Artificial Intelligence for Quantitative Modeling in Drug Discovery and Development: An Innovation and Quality Consortium Perspective on Use Cases and Best Practices. Clin. Pharmacol. Ther..

[172] Staszak M., Staszak K., Wieszczycka K., Bajek A., Roszkowski K., Tylkowski B. (2022). Machine learning in drug design: Use of artificial intelligence to explore the chemical structure–biological activity relationship. WIREs Comput. Mol. Sci..

[173] Selvaraj C., Chandra I., Singh S.K. (2022). Artificial intelligence and machine learning approaches for drug design: Challenges and opportunities for the pharmaceutical industries. Mol. Divers..

[174] Spitz S., Zhang Y., Fischer S., McGuire K., Sommer U., Amaravadi L., Bandukwala A., Eck S., Garofolo F., Islam R. (2021). 2020 White Paper on Recent Issues in Bioanalysis: BAV Guidance, CLSI H62, Biotherapeutics Stability, Parallelism Testing, CyTOF and Regulatory Feedback (Part 2A—Recommendations on Biotherapeutics Stability, PK LBA Regulated Bioanalysis, Biomarkers Assays, Cytometry Validation & Innovation Part 2B—Regulatory Agencies’ Inputs on Bioanalysis, Biomarkers, Immunogenicity, Gene & Cell Therapy and Vaccine). Bioanalysis.

[175] Jose J., Shifali S., Mathew B., Parambi D.G.T. (2022). In Silico Trial Approach for Biomedical Products: A Regulatory Perspective. Comb. Chem. High Throughput Screen..

[176] Deshmukh A., Goyal R., Sundaram K., Dange K., Lakhote T., Niranjan S., Bharucha J., Mishra A., Vats B., Tiwari S. (2023). Analytical sameness methodology for the evaluation of structural, physicochemical, and biological characteristics of Armlupeg: A pegfilgrastim biosimilar case study. PLoS ONE.

[177] Rahalkar H., Cetintas H.C., Salek S. (2018). Quality, Non-clinical and Clinical Considerations for Biosimilar Monoclonal Antibody Development: EU, WHO, USA, Canada, and BRICS-TM Regulatory Guidelines. Front. Pharmacol..

[178] Zagalo D.M., Sousa J., Simões S. (2022). Quality by design (QbD) approach in marketing authorization procedures of Non-Biological Complex Drugs: A critical evaluation. Eur. J. Pharm. Biopharm..

[179] Wolff-Holz E., Tiitso K., Vleminckx C., Weise M. (2019). Evolution of the EU Biosimilar Framework: Past and Future. BioDrugs.

[180] Zhang R., Li X., Zhang X., Qin H., Xiao W. (2021). Machine learning approaches for elucidating the biological effects of natural products. Nat. Prod. Rep..

[181] Kovács B., Péterfi O., Kovács-Deák B., Székely-Szentmiklósi I., Fülöp I., Bába L.I., Boda F. (2021). Quality-by-design in pharmaceutical development: From current perspectives to practical applications. Acta Pharm..

[182] Mohseni-Motlagh S.F., Dolatabadi R., Baniassadi M., Baghani M. (2023). Application of the Quality by Design Concept (QbD) in the Development of Hydrogel-Based Drug Delivery Systems. Polymers.

[183] Pardeshi S.R., Deshmukh N.S., Telange D.R., Nangare S.N., Sonar Y.Y., Lakade S.H., Harde M.T., Pardeshi C.V., Gholap A., Deshmukh P.K. (2023). Process development and quality attributes for the freeze-drying process in pharmaceuticals, biopharmaceuticals and nanomedicine delivery: A state-of-the-art review. Future J. Pharm. Sci..

[184] Forbes T.P., Gillen J.G., Feeney W., Ho J. (2024). Quality by Design Considerations for Drop-on-Demand Point-of-Care Pharmaceutical Manufacturing of Precision Medicine. Mol. Pharm..

[185] Castel J., Delaux S., Hernandez-Alba O., Cianférani S. (2023). Recent advances in structural mass spectrometry methods in the context of biosimilarity assessment: From sequence heterogeneities to higher order structures. J. Pharm. Biomed. Anal..

[186] Biehn S.E., Lindert S. (2022). Protein Structure Prediction with Mass Spectrometry Data. Annu. Rev. Phys. Chem..

[187] Fukushima Y., Yoshidome T. (2023). A deep-learning model for grid-based solvation free energy. Biophys. J..

[188] Muzio G., O’Bray L., Borgwardt K. (2021). Biological network analysis with deep learning. Briefings Bioinform..

[189] Vora L.K., Gholap A.D., Jetha K., Thakur R.R.S., Solanki H.K., Chavda V.P. (2023). Artificial Intelligence in Pharmaceutical Technology and Drug Delivery Design. Pharmaceutics.

[190] Borjali A., Magnéli M., Shin D., Malchau H., Muratoglu O.K., Varadarajan K.M. (2021). Natural language processing with deep learning for medical adverse event detection from free-text medical narratives: A case study of detecting total hip replacement dislocation. Comput. Biol. Med..

[191] McComb M., Bies R., Ramanathan M. (2022). Machine learning in pharmacometrics: Opportunities and challenges. Br. J. Clin. Pharmacol..

[192] Xue X., Qian J. (2024). Safety of marketed biosimilar monoclonal antibody cancer treatments in the US: A disproportionality analysis using the food and drug administration adverse event reporting system (FAERS) database. Expert Opin. Drug Saf..

[193] Xue X., Truong B., Qian J. (2023). Adverse event reporting of marketed biosimilar and biological monoclonal antibody cancer treatments in the United States. Expert Opin. Biol. Ther..

[194] Batko K., Ślęzak A. (2022). The use of Big Data Analytics in healthcare. J. Big Data.

[195] Lindroth H., Nalaie K., Raghu R., Ayala I.N., Busch C., Bhattacharyya A., Moreno Franco P., Diedrich D.A., Pickering B.W., Herasevich V. (2024). Applied Artificial Intelligence in Healthcare: A Review of Computer Vision Technology Application in Hospital Settings. J. Imaging.

[196] Mascarenhas-Melo F., Diaz M., Gonçalves M.B.S., Vieira P., Bell V., Viana S., Nunes S., Paiva-Santos A.C., Veiga F. (2024). An Overview of Biosimilars—Development, Quality, Regulatory Issues, and Management in Healthcare. Pharmaceuticals.

[197] Kwon M., Joung C.I., Shin H., Lee C.C., Song Y.S., Lee Y.J., Kang S., Kim J.Y., Lee S. (2024). Detection of novel drug-adverse drug reaction signals in rheumatoid arthritis and ankylosing spondylitis: Analysis of Korean real-world biologics registry data. Sci. Rep..

[198] Malikova M.A. (2020). Practical applications of regulatory requirements for signal detection and communications in pharmacovigilance. Ther. Adv. Drug Saf..

[199] Alowais S.A., Alghamdi S.S., Alsuhebany N., Alqahtani T., Alshaya A.I., Almohareb S.N., Aldairem A., Alrashed M., Bin Saleh K., Badreldin H.A. (2023). Revolutionizing healthcare: The role of artificial intelligence in clinical practice. BMC Med. Educ..

[200] Kawasaki R. (2024). How Can Artificial Intelligence Be Implemented Effectively in Diabetic Retinopathy Screening in Japan?. Medicina.

[201] Mariam Z., Niazi S.K., Magoola M. (2024). Unlocking the Future of Drug Development: Generative AI, Digital Twins, and Beyond. BioMedInformatics.

[202] Ang D., Rakovski C., Atamian H.S. (2024). De Novo Drug Design Using Transformer-Based Machine Translation and Reinforcement Learning of an Adaptive Monte Carlo Tree Search. Pharmaceuticals.

[203] Grechishnikova D. (2021). Transformer neural network for protein-specific de novo drug generation as a machine translation problem. Sci. Rep..

[204] Chiu K., Racz R., Burkhart K., Florian J., Ford K., Iveth Garcia M., Geiger R.M., Howard K.E., Hyland P.L., Ismaiel O.A. (2023). New science, drug regulation, and emergent public health issues: The work of FDA’s division of applied regulatory science. Front. Med..

[205] Liu C., Cheng S., Chen C., Qiao M., Zhang W., Shah A., Bai W., Arcucci R. (2023). M-FLAG: Medical Vision-Language Pre-training with Frozen Language Models and Latent Space Geometry Optimization. Proceedings of the Medical Image Computing and Computer Assisted Intervention—MICCAI.

[206] Li J., Liu C., Cheng S., Arcucci R., Hong S. Frozen Language Model Helps ECG Zero-Shot Learning. Proceedings of the Medical Imaging with Deep Learning.

[207] Wan Z., Liu C., Zhang M., Fu J., Wang B., Cheng S., Ma L., Quilodrán-Casas C., Arcucci R. (2023). Med-UniC: Unifying Cross-Lingual Medical Vision-Language Pre-Training by Diminishing Bias. arXiv.

[208] Hair J., Maryon T., Lieneck C. (2022). Identification of Barriers Preventing Biosimiliar Oncology Medication Adoption. Medicina.

[209] Joshi D., Khursheed R., Gupta S., Wadhwa D., Singh T.G., Sharma S., Porwal S., Gauniyal S., Vishwas S., Goyal S. (2022). Biosimilars in Oncology: Latest Trends and Regulatory Status. Pharmaceutics.

[210] Teng F., Cui T., Zhou L., Gao Q., Zhou Q., Li W. (2024). Programmable synthetic receptors: The next-generation of cell and gene therapies. Signal Transduct. Target. Ther..

[211] Arman S., Tilley R.D., Gooding J.J. (2024). A review of electrochemical impedance as a tool for examining cell biology and subcellular mechanisms: Merits, limits, and future prospects. Analyst.

[212] Foglizzo V., Marchiò S. (2022). Nanoparticles as Physically- and Biochemically-Tuned Drug Formulations for Cancers Therapy. Cancers.

[213] Fong E.J., Strelez C., Mumenthaler S.M. (2020). A Perspective on Expanding Our Understanding of Cancer Treatments by Integrating Approaches from the Biological and Physical Sciences. SLAS Discov..

[214] Lorenzo G., Ahmed S.R., Hormuth D.A., Vaughn B., Kalpathy-Cramer J., Solorio L., Yankeelov T.E., Gomez H. (2023). Patient-specific, mechanistic models of tumor growth incorporating artificial intelligence and big data. arXiv.

[215] Fujita K.i., Matsumoto N., Ishida H., Kubota Y., Iwai S., Shibanuma M., Kato Y. (2019). Decreased Disposition of Anticancer Drugs Predominantly Eliminated via the Liver in Patients with Renal Failure. Curr. Drug Metab..

[216] Malinzi J., Basita K.B., Padidar S., Adeola H.A. (2021). Prospect for application of mathematical models in combination cancer treatments. Inform. Med. Unlocked.

[217] Delrue C., Speeckaert M.M. (2022). The Potential Applications of Raman Spectroscopy in Kidney Diseases. J. Pers. Med..

[218] Amasawa E., Kuroda H., Okamura K., Badr S., Sugiyama H. (2021). Cost–Benefit Analysis of Monoclonal Antibody Cultivation Scenarios in Terms of Life Cycle Environmental Impact and Operating Cost. ACS Sustain. Chem. Eng..

[219] Bittner B. (2023). Customer-centric product presentations for monoclonal antibodies. AAPS Open.

[220] Fan X., Krzyzanski W., Wong R.S.M., Liu D., Yan X. (2023). Novel Combination of Erythropoietin and Romiplostim to Treat Chemotherapy-Induced Anemia and Thrombocytopenia via Pharmacodynamic Interaction on Hematopoietic Stem and Progenitor Cells. ACS Pharmacol. Transl. Sci..

[221] Gilaberte Reyzabal S., Isenberg D. (2022). Differences in the Development of Adverse Infusion Reactions to Rituximab in Patients With Systemic Lupus Erythematosus, Rheumatoid Arthritis and Non-Hodgkin’s Lymphoma-Enigma Variations. Front. Med..

[222] Peipert J.D., Kaiser K., Kircher S., Greene G.J., Shaunfield S., Hauner K., Cella D., Mroczek D.K. (2023). Medical Oncologists’ Knowledge and Perspectives on the Use of Biosimilars in the United States. JCO Oncol. Pract..

[223] Evangelatos G., Bamias G., Kitas G.D., Kollias G., Sfikakis P.P. (2022). The second decade of anti-TNF-a therapy in clinical practice: New lessons and future directions in the COVID-19 era. Rheumatol. Int..

[224] Cliff E.R.S., Rome R.S., Kesselheim A.S., Rome B.N. (2023). National Comprehensive Cancer Network Guideline Recommendations of Cancer Drugs With Accelerated Approval. JAMA Netw. Open.

[225] Gherghescu I., Delgado-Charro M.B. (2020). The Biosimilar Landscape: An Overview of Regulatory Approvals by the EMA and FDA. Pharmaceutics.

[226] Kvien T.K., Patel K., Strand V. (2022). The cost savings of biosimilars can help increase patient access and lift the financial burden of health care systems. Semin. Arthritis Rheum..

[227] Greene L., Singh R.M., Carden M.J., Pardo C.O., Lichtenstein G.R. (2019). Strategies for Overcoming Barriers to Adopting Biosimilars and Achieving Goals of the Biologics Price Competition and Innovation Act: A Survey of Managed Care and Specialty Pharmacy Professionals. J. Manag. Care Spec. Pharm..

[228] Lengyel C.G., Habeeb B.S., Altuna S.C., Trapani D., Khan S.Z., Hussain S., Al Jarroudi O., El Bairi K., Curigliano G. (2023). The Global Landscape on the Access to Cancer Medicines for Breast Cancer: The ONCOLLEGE Experience. Breast Cancer Research and Treatment.

[229] Wang L., Chen X., Zhang L., Li L., Huang Y., Sun Y., Yuan X. (2023). Artificial intelligence in clinical decision support systems for oncology. Int. J. Med. Sci..

[230] Mysler E., Azevedo V.F., Danese S., Alvarez D., Iikuni N., Ingram B., Mueller M., Peyrin-Biroulet L. (2021). Biosimilar-to-Biosimilar Switching: What is the Rationale and Current Experience?. Drugs.

[231] Nicoletti M.M., Crisci E., Pentella C., Cantone A., Ruggiero D., Anatriello A., Scavone C. (2023). Switching between Originators and Biosimilars in Dermatology: A Systematic Review of Real-World Clinical Studies. Biologics.

[232] Wirth K., Boes S., Näpflin M., Huber C., Blozik E. (2023). Initial prescriptions and medication switches of biological products: An analysis of prescription pathways and determinants in the Swiss healthcare setting. BMJ Open.

[233] Liu R., Wang L., Rizzo S., Garmhausen M.R., Pal N., Waliany S., McGough S., Lin Y.G., Huang Z., Neal J. (2024). Systematic analysis of off-label and off-guideline cancer therapy usage in a real-world cohort of 165,912 US patients. Cell Rep. Med..

[234] Iqbal Z., Sadaf S. (2022). Biosimilars: A Comparative Study of Regulatory, Safety and Pharmacovigilance Monograph in the Developed and Developing Economies. J. Pharm. Pharm. Sci..

[235] Barbier L., Simoens S., Vulto A.G., Huys I. (2020). European Stakeholder Learnings Regarding Biosimilars: Part I—Improving Biosimilar Understanding and Adoption. BioDrugs.

[236] Florio M., Gamba S. (2021). Biomed Europa: After the coronavirus, a public infrastructure to overcome the pharmaceutical oligopoly. Ann. Public Coop. Econ..

[237] Feldman R. (2023). Trade Secrets in Biologic Medicine: The Boundary with Patents. Sci. Technol. Law Rev..

[238] Rahalkar H., Sheppard A., Santos G.M.L., Dasgupta C., Perez-Tapia S.M., Lopez-Morales C.A., Salek S. (2021). Current Regulatory Requirements for Biosimilars in Six Member Countries of BRICS-TM: Challenges and Opportunities. Front. Med..

[239] Mercurio B., Upreti P.N. (2023). Patent term extension and test data protection obligations: Identifying the gap in policy, research, and practice of implementing free trade agreements. J. Law Biosci..

[240] Simoens S. (2021). How do biosimilars sustain value, affordability, and access to oncology care?. Expert Rev. Pharmacoecon. Outcomes Res..

[241] Hübel K., Kron F., Lux M.P. (2020). Biosimilars in oncology: Effects on economy and therapeutic innovations. Eur. J. Cancer.

[242] Nahleh Z., Lyman G.H., Schilsky R.L., Peterson D.E., Tagawa S.T., Chavez-MacGregor M., Rumble R.B., Gupta S. (2022). Use of Biosimilar Medications in Oncology. JCO Oncol. Pract..

[243] García J.J., Raez L.E., Rosas D. (2020). A narrative review of biosimilars: A continued journey from the scientific evidence to practice implementation. Transl. Lung Cancer Res..

[244] Kurki P., Barry S., Bourges I., Tsantili P., Wolff-Holz E. (2021). Safety, Immunogenicity and Interchangeability of Biosimilar Monoclonal Antibodies and Fusion Proteins: A Regulatory Perspective. Drugs.

[245] Khan M.A.A.K., Turjya R.R., Islam A.B.M.M.K. (2021). Computational engineering the binding affinity of Adalimumab monoclonal antibody for designing potential biosimilar candidate. J. Mol. Graph. Model..

[246] Kute N., Mankar S.D., Bhawar S.B. (2022). Biosimilar and it’s Current Perspective—A Review. Res. J. Pharmacol. Pharmacodyn..

[247] Blackstone E.A., Joseph P.F. (2013). The Economics of Biosimilars. Am. Health Drug Benefits.

[248] Da Silva R.G.L. (2024). The advancement of artificial intelligence in biomedical research and health innovation: Challenges and opportunities in emerging economies. Glob. Health.

[249] Cortes J., Perez-García J.M., Llombart-Cussac A., Curigliano G., El Saghir N.S., Cardoso F., Barrios C.H., Wagle S., Roman J., Harbeck N. (2020). Enhancing global access to cancer medicines. CA Cancer J. Clin..

[250] Kar I., Kronz M., Kolychev E., Silverman P., Mendiratta P., Tomlinson B.K.N., Prunty J., Copley M., Patel S., Caudill S. (2022). Biosimilar strategic implementation at a large health system. Am. J. Health-Syst. Pharm..

[251] Bond A.M., Dean E.B., Desai S.M. (2023). The Role of Financial Incentives in Biosimilar Uptake in Medicare: Evidence from the 340B Program: Study examines the role of financial incentives in the uptake of biosimilar drugs in Medicare. Health Aff..

[252] Lam S.W., Amoline K., Marcum C., Leonard M. (2021). Healthcare system conversion to a biosimilar: Trials and tribulations. Am. J. Health-Syst. Pharm..

